# A Mechanistic Computational Model Reveals That Plasticity of CD4^+^ T Cell Differentiation Is a Function of Cytokine Composition and Dosage

**DOI:** 10.3389/fphys.2018.00878

**Published:** 2018-08-02

**Authors:** Bhanwar Lal Puniya, Robert G. Todd, Akram Mohammed, Deborah M. Brown, Matteo Barberis, Tomáš Helikar

**Affiliations:** ^1^Department of Biochemistry, University of Nebraska–Lincoln, Lincoln, NE, United States; ^2^Department of Natural and Applied Sciences, Mount Mercy University, Cedar Rapids, IA, United States; ^3^School of Biological Sciences, University of Nebraska–Lincoln, Lincoln, NE, United States; ^4^Nebraska Center for Virology, University of Nebraska–Lincoln, Lincoln, NE, United States; ^5^Synthetic Systems Biology and Nuclear Organization, Swammerdam Institute for Life Sciences, University of Amsterdam, Amsterdam, Netherlands; ^6^Molecular Cell Physiology, VU University Amsterdam, Amsterdam, Netherlands

**Keywords:** CD4^+^ T cell differentiation, T cell plasticity, complex T cell phenotypes, regulation of T cell plasticity, cytokine compositions, cytokine dosage

## Abstract

CD4^+^ T cells provide cell-mediated immunity in response to various antigens. During an immune response, naïve CD4^+^ T cells differentiate into specialized effector T helper (Th1, Th2, and Th17) cells and induced regulatory (iTreg) cells based on a cytokine milieu. In recent studies, complex phenotypes resembling more than one classical T cell lineage have been experimentally observed. Herein, we sought to characterize the capacity of T cell differentiation in response to the complex extracellular environment. We constructed a comprehensive mechanistic (logical) computational model of the signal transduction that regulates T cell differentiation. The model’s dynamics were characterized and analyzed under 511 different environmental conditions. Under these conditions, the model predicted the classical as well as the novel complex (mixed) T cell phenotypes that can co-express transcription factors (TFs) related to multiple differentiated T cell lineages. Analyses of the model suggest that the lineage decision is regulated by both compositions and dosage of signals that constitute the extracellular environment. In this regard, we first characterized the specific patterns of extracellular environments that result in novel T cell phenotypes. Next, we predicted the inputs that can regulate the transition between the canonical and complex T cell phenotypes in a dose-dependent manner. Finally, we predicted the optimal levels of inputs that can simultaneously maximize the activity of multiple lineage-specifying TFs and that can drive a phenotype toward one of the co-expressed TFs. In conclusion, our study provides new insights into the plasticity of CD4^+^ T cell differentiation, and also acts as a tool to design testable hypotheses for the generation of complex T cell phenotypes by various input combinations and dosages.

## Introduction

The diversity and number of immunity-related diseases require a high level of heterogeneity in the immune system to maintain the overall well-being of a human. Early studies of immune responses led to a discovery that the CD4^+^ T cells (referred to as T cells), which are critical players in immunity, can be classified into two subtypes - T helper 1 (Th1) and T helper 2 (Th2) cells (Mosmann et al., 1986). Each type of effector T cell produces a specific set of cytokines that define the function of the cell and the way it further governs the immune response. Specifically, the Th1 cells are responsible for several autoimmune diseases, whereas the Th2 cells are the mediators in cases of allergy and asthma (Reiner, 2007; Zhu and Paul, 2008). More recently, a number of additional T cell subtypes, including the inducible regulatory T cells (iTregs) (Groux et al., 1997; Chen et al., 2003; Schmitt and Williams, 2013), T helper 17 (Th17) (Romagnani, 2000; Harrington et al., 2005; Mangan et al., 2006), T helper 9 (Th9) (Dardalhon et al., 2008; Veldhoen et al., 2008; Soroosh and Doherty, 2009), and follicular T helper cells (Tfh) (Breitfeld et al., 2000; Schaerli et al., 2000) have been discovered, and their functions have been extensively studied. For example, the Th17 cells have been found to be responsible for assisting the immune response against extracellular bacteria and fungi, whereas the main role of the iTregs is to maintain the balance and regulate immune responses by the T helper cell subtypes (Zhu and Paul, 2008). The Th9 cells have been found to be involved in pathogen immunity and inflammatory diseases (Kaplan, 2013). Finally, the Tfh cells assist in T cell-dependent B cell response (Breitfeld et al., 2000; Schaerli et al., 2000; Ma et al., 2012).

In addition, recent studies suggest that some T helper cells are capable of switching and exhibiting phenotypes of one of the alternative effector T cells, depending on the combination of input signals that the cell receives. For example, the iTregs and Th17 can switch from one phenotype to the other in response to the pleiotropic cytokine interleukin-6 (IL-6) (Xu et al., 2007; Lee et al., 2009a; Rowell and Wilson, 2009; Kimura and Kishimoto, 2010). The fully differentiated Th17 cells have been observed to produce Th1-cell-specific cytokines (Shi et al., 2008; Lee et al., 2009b; Nindl et al., 2012; Harbour et al., 2015). The Th2 cells have been reported to further develop into Th9 cells (Veldhoen et al., 2008). More complexity in T cell differentiation was observed in the form of co-expression of mutually exclusive lineage-specifying transcription factors (TFs) (Peine et al., 2013; Bock et al., 2017). This co-expression can lead to the development of stable or intermediate subtypes that share characteristics of more than one type of T cell (Tartar et al., 2010). Examples of such mixed (complex) phenotypes include Th1–Th2 (Peine et al., 2013; Bock et al., 2017) and Th1–Th17 (Kullberg et al., 2006; Morrison et al., 2013).

The differentiation process is governed by the regulation of multiple, mutually cross-linked signaling pathways, which form complex networks (Zhu et al., 2010). The stimulation of the naive CD4^+^ T cells by various cytokines triggers a cascade of signaling events, such as the activation of the JAK/STAT pathways that lead to the activation of T cell lineage-specifying TFs (Murphy and Reiner, 2002; Kaiko et al., 2008). For example, the commitment to Th1 lineage is initiated through signaling by interferon gamma (IFN-γ) and IL-12, leading to the activation of STAT1/STAT4, which in turn activate the T box expressed in T cells (Tbet). Differentiation into Th2 is stimulated by the activation of the GATA binding protein 3 (GATA3) TF through STAT6 signaling. The differentiation of naive T cells into Th17 is governed by the retinoic acid receptor-related orphan receptor gamma t (RORγt) TF, and by the cytokines i.e., IL-6, IL-21, IL-23 and the transforming growth factor beta (TGF-β) (Aggarwal et al., 2003; Harrington et al., 2005; Park et al., 2005; Tesmer et al., 2008). In addition, the TGF-β inhibits T cell differentiation to both the Th1 and Th2 lineages and is also conducive to the cell’s commitment to the iTregs lineage (Schmitt and Williams, 2013).

The complexity of biochemical networks underlying the regulation of T cell differentiation leads to additional questions regarding the mechanisms of the immune response. For instance, based on a large number of possible combinations of extracellular cues, we may ask the following questions: (i) How does the cell decide into which subsequent lineage to differentiate? (ii) What specific combinations of signals are driving a possible switch to a different lineage? (iii) What specific mechanisms are responsible for the T cell differentiation capacity and plasticity?

While regulation of T cell differentiation in the context of the diverse cytokine microenvironment has been studied extensively, effects of the interplay among multiple cytokines on T cell differentiation remain an open question. A systems-level computational model can be used to explore whether, and to what extent, the extracellular cytokine milieu affects the T cell differentiation program. Recently, computational models using various types of mathematical approaches investigated the regulation of phenotypic plasticity, and dynamics in response to diseases (Naldi et al., 2010; Carbo et al., 2013, 2014; Abou-Jaoudé et al., 2014; Martinez-Sanchez et al., 2015). Predictions from these models include novel T cell differentiation pathways (Naldi et al., 2010), transition among T cell types under various micro-environments and perturbations (Martinez-Sanchez et al., 2015), peroxisome proliferator-activated receptor gamma-dependent regulation of Th17 to iTreg switch (Carbo et al., 2013), and IL-21-dependent modulation of IL-10 (Carbo et al., 2014).

Here, we explored the effect of the interplay among extracellular cytokines on differentiation of T cells and their plasticity. We have developed a logic-based computational model (Helikar and Rogers, 2009; Helikar et al., 2012a,b, 2013; Naldi et al., 2015; Abou-Jaoudé et al., 2016; Barberis et al., 2017; Linke et al., 2017) of a signal transduction network that regulates the differentiation process of naive T cells to Th1, Th2, Th17, and iTreg cells and analyzed its dynamics. Local protein–protein regulatory information was manually curated to construct the mechanistic model that contains lineage-specifying TFs (Tbet, GATA3, RORγt, and Foxp3), various signal transducers and activators of transcription (STATs), and other signaling molecules. The model consists of 96 regulatory interactions among 38 components. To explore the entire cytokine microenvironment, we analyzed the model’s dynamics under (i) all possible combinations of extracellular signals, and (ii) various input dosages. The analysis of the model resulted in dynamic signatures that represent previously described, as well as novel cellular phenotypes. These include four canonical phenotypes of differentiated T cells (Th0, Th1, Th2, and iTreg) as well as six complex phenotypes, whereby multiple lineage-specifying TFs are co-expressed. Our results also suggest that the input dosage regulates the balance of specific T cells within the complex T cell phenotypes, providing new insights into specific patterns of environmental input composition and dosage effects on T cell differentiation.

## Results

### Mechanistic Logical Model of T Cell Differentiation

**FIGURE 1:**
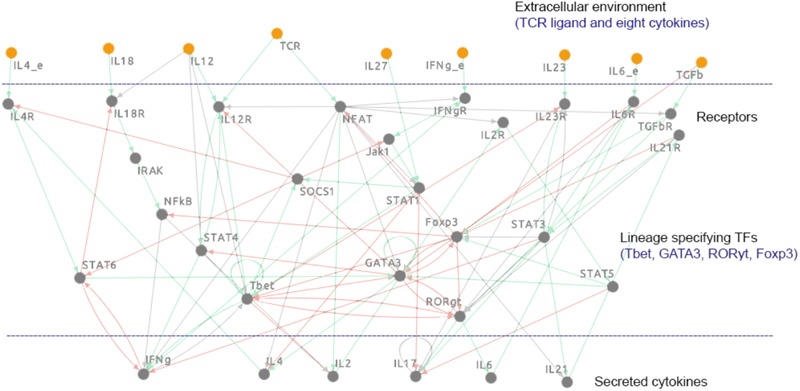
Network diagram of the logical model of signal transduction in CD4^+^ T cells. The modeled pathways reflect the canonical pathways known to regulate T cell differentiation into the major effector subtypes (Th1, Th2, Th17) and the regulatory subtype (iTreg). The model includes 38 components, including four lineage-specifying TFs (Tbet, GATA3, RORγt, and Foxp3) and nine extracellular components: TCR-ligand, IFN-γ, TGF-β, IL-4, IL-6, IL-12, IL-18, IL-23, and IL-27. Green arrows represent activation, red arrows represent inhibition, and gray arrows represent the conditions associated with activatory or inhibitory interactions. IFNg_e, interferon-γ (external); IL12, interleukin 12; IL18, interleukin 18; IL23, interleukin 23; IL27, interleukin 27; IL4_e, interleukin 4 (external); IL6_e, interleukin 6 (external); TCR, T cell receptor; TGFb, transforming growth factor-β; Foxp3, Forkhead box P3; GATA3, GATA-binding protein 3; IFNg, interferon-γ; IFNgR, interferon-γ receptor (generic); IL12R, interleukin 12 receptor (generic); IL17, interleukin 17; IL18R, interleukin 18 receptor 1; IL2, interleukin 2; IL21, interleukin 21; IL21R, interleukin 21 receptor; IL23R, interleukin 23 receptor; IL2R, interleukin 2 receptor; IL4, interleukin 4; IL4R, interleukin 4 receptor; IL6, interleukin 6; IL6R, interleukin 6 receptor; IRAK, interleukin-1 receptor associated kinase 1; Jak1, Janus kinase 1; NFAT, nuclear factor of activated T cells 5, tonicity-responsive; NF-κB, nuclear factor of kappa light polypeptide gene enhancer in B cells (generic); RORgt, RAR-related orphan receptor C; SOCS1, suppressor of cytokine signaling 1; STAT1, signal transducer and activator 1; STAT3, signal transducer and activator 3; STAT4, signal transducer and activator 4; STAT5, signal transducer and activator 5; STAT6, signal transducer and activator 6; Tbet, T-box expressed in T cells; TGFbR, transforming growth factor-β receptor (generic).

A comprehensive mechanistic, logic-based model of T cell differentiation was constructed using regulatory information from published literature. The model includes 38 components and 96 biochemical interactions that regulate the differentiation process of major T cell subtypes, such as Th1, Th2, Th17, and iTreg cells. The individual components of the model represent lineage-specifying TFs (Tbet, GATA3, RORγt, and Foxp3), STAT proteins, cytokines, their receptors, and other signaling molecules. The extracellular environment is represented in the model by eight cytokines and a (generic) TCR ligand, known to play a role in T cell differentiation. The network representation of the model is visualized in **Figure 1**. The regulatory interactions in the model are defined as Boolean functions, which are composed of the “AND,” “OR,” and “NOT” operators (Supplementary Datasheets 1 and 2). The fully annotated model is available for download in a number of formats [including SBML-qual (Chaouiya et al., 2013)], as well as for viewing, and performing simulations, analyses, and additional modifications within the Cell Collective modeling platform^1^ (Helikar et al., 2012b, 2013). The model can be accessed directly at: https://www.cellcollective.org/#6678/cd4-t-cell-differentiation.

**FIGURE 2:**
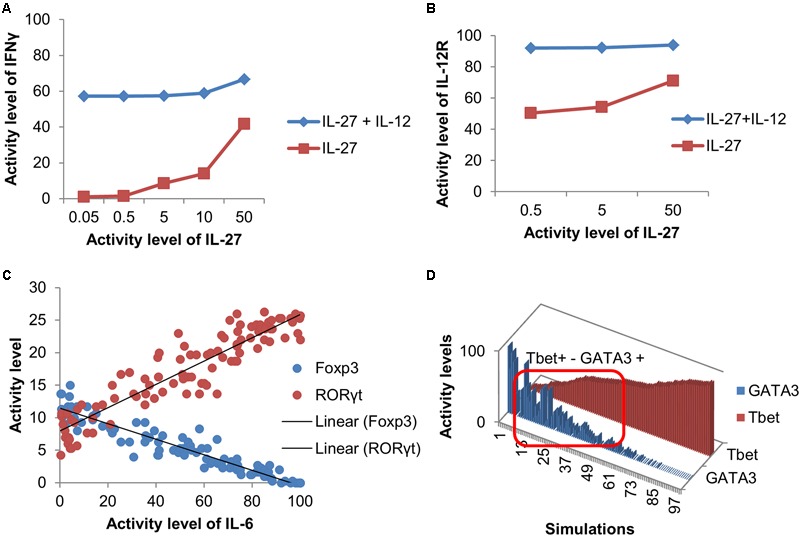
Validation of the model using complex behaviors. **(A–B)** Simulations of the model under experimental conditions using the same concentration ratio of IL-27 and IL-12 (i.e., 1000, 100, 10, 1 for IFN-γ and 100, 10, 1 for IL-12R) as in Becskei and Grusby (2007) show that combination of IL-27 with IL-12 leads to a synergistic effect on level of IFN-γ production and the activity of the IL-12 receptor (IL-12R). **(C)** Simulated IL-6 dose-response effect on the activation of Foxp3 and RORγt. **(D)** Model simulations reproduce a mixed Th1/Th2 phenotype with varying levels of Tbet and GATA3. The significant activity levels of both Tbet and GATA3 are observed in the area inside the red box.

The model was validated to ensure that it can reproduce differentiation into four canonical phenotypes (Th1, Th2, Th17, and iTreg), as a result of cytokine stimulation and TCR activation (Supplementary Table 1). Furthermore, the model was able to reproduce more complex behaviors (**Figure 2**). For example, Becskei and Grusby (2007) studied the synergistic effect of positive feedback loops on the expression of the IL-12 receptor (IL-12R). They showed that the number of IFN-γ positive cells and the expression of IL-12R increased when induced by the combination of IL-12 and IL-27. As shown in **Figures 2A,B**, simulations of the presented model under similar experimental conditions resulted in the same qualitative behavior. Furthermore, it has been experimentally shown that the IL-6 regulates the balance between iTreg and Th17 cells in a dose-dependent manner (Yang et al., 2008; Kimura and Kishimoto, 2010). Similarly, simulations of the model show a clear distinction between iTreg and Th17 in an IL-6-dependent manner (**Figure 2C**). Finally, simulations of the model, under environmental conditions similar to those that have been shown to induce the mixed Th1–Th2 behavior (Peine et al., 2013), also resulted in a complex phenotype with activation of both Tbet and GATA3 TFs (**Figure 2D**).

### Novel T Cell Phenotypes Are Predicted by Logical Modeling

**FIGURE 3:**
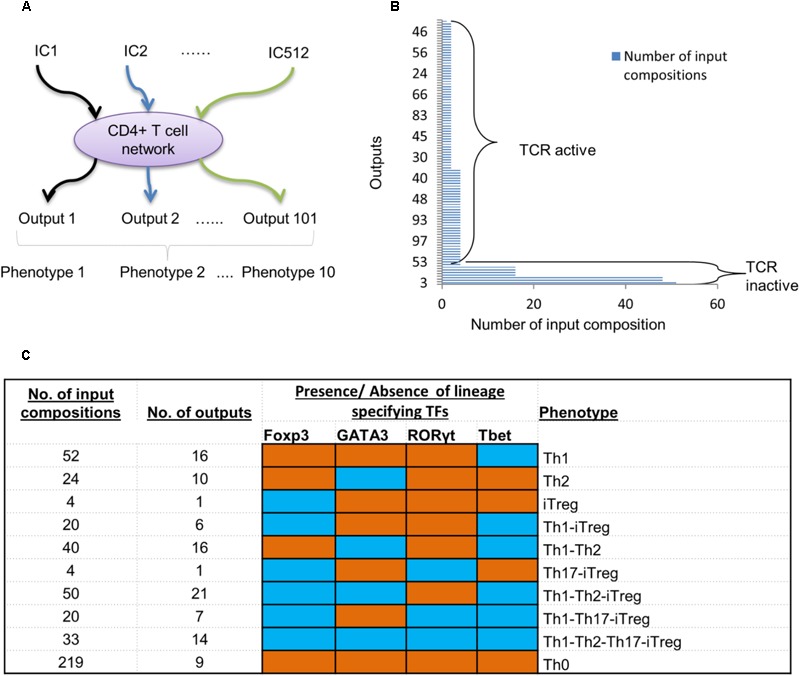
Simulation of T cell model under all possible environmental conditions. **(A)** A schematic diagram showing simulation strategy using all possible input compositions (IC). **(B)** A total of 101 outputs (ergodic sets) were obtained. The number of input compositions stimulating each ergodic set is ranging from lowest 1 to highest 51. Only one ergodic set resulted from a single input composition. **(C)** Ten phenotypes based on presence and absence of lineage-specifying TFs were obtained. The number of input compositions and ergodic sets for each phenotype are provided in the first and second column. Based on the presence and absence of TFs, each phenotype was determined. Blue cells correspond to TFs present in the phenotype, whereas the orange cells represent inactive TFs.

With the validated model in hand, we sought to understand its capacity to represent various T cell phenotypes. By using ergodic set analysis [see the section “Materials and Methods” and Todd and Helikar (2012)], we explored the state space of the model under 512 possible combinations of the extracellular stimuli (*input compositions*) (**Figure 3A**).

A total of 101 ergodic sets (*outputs*) were obtained as a result of 511 input compositions (Supplementary Table 2). Out of the 511 compositions, 45 input compositions resulted into fixed points (a single remaining input composition was not analyzable even on a supercomputer due to the large size of state space which could not be computed on a feasible temporal scale). The number of input compositions for each output ranged from 1 to 51 (**Figure 3B**). We obtained one output (output 3) that can be stimulated by the maximum of 51 input compositions. Two outputs (outputs 6 and 13) were each stimulated by the maximum of 48 input compositions (**Figure 3B**). Furthermore, four outputs (outputs 10, 22, 29, and 32) were each achieved by 16 different input compositions. All outputs that are individually stimulated by 16 or more input compositions have input compositions with an inactive TCR ligand.

The number of input compositions for the remaining outputs varied from 1 to 4. These input compositions contained an active TCR ligand. In this group, a total of 37 outputs were obtained, whereby each of them was stimulated by four input compositions. A total of 56 outputs were each stimulated with two input compositions. Only one output was stimulated by a single input composition.

Thus, 7 (out of 101) outputs were achieved when stimulated by 211 input compositions with the absence of a TCR ligand. On the other hand, 94 outputs (out of 101) were obtained when stimulated by 255 input compositions with an active TCR ligand. Therefore, fewer outputs (101) have been observed than the total number of input compositions (511), suggesting that a specific cell fate (output) can result from multiple signal compositions, processed by a cell based on biochemical rules in a signaling network (Helikar et al., 2008; Balázsi et al., 2011; Palau-Ortin et al., 2015).

Next, we explored the biological relevance of the produced outputs. As the model centers on the regulation of T cell phenotypes and the TFs related to each differentiated T cell subtype, we classified all the outputs based on the presence of the four TFs (GATA3, Tbet, RORγt, and Foxp3). We found that the model outputs (as a result of the 511 input compositions) cluster into 10 biologically relevant phenotypes. These include the canonical (single cell type) phenotypes as well as the complex phenotypes having more than one lineage-specifying TF.

Specifically, we found four canonical T cell phenotypes that carried Tbet, GATA3, or Foxp3, representing Th1, Th2, and iTreg, respectively (**Figure 3C**). Furthermore, we found that 219 input compositions resulted in nine outputs with no TFs present (Th0 phenotype). Most of the outputs that represent the Th0 phenotype (>95%) were stimulated by the input compositions with an inactive TCR ligand. The remaining Th0-leading input compositions contained an active TCR ligand along with IL-23, or IL-18, or IL-6. This corresponds to the experimentally established scenarios, whereby the T cells cannot differentiate in the absence of TCR activation or in the absence of key lineage-specific cytokines (Podojil and Miller, 2009; Zhu et al., 2010; Chen and Flies, 2013). Fifty-two input compositions led to 16 outputs with active Tbet, representing the Th1 phenotype. A total of 24 input compositions produce 10 outputs with active GATA3, representing the Th2 phenotype, while four input compositions led to one output with active Foxp3, representing the iTreg phenotype. We did not observe distinct outputs with only RORγt active; instead, RORγt was part of the complex phenotypes (discussed below).

In addition to the four canonical phenotypes, the model predicted six complex phenotypes. The number of input compositions, and the number of outputs that represent each complex phenotype, is summarized in **Figure 3C**. Of the six complex phenotypes, three of them including Th1–Th2 (Hegazy et al., 2010; Evans and Jenner, 2013; Peine et al., 2013), Th1–iTreg (Koch et al., 2009), and Th17–iTreg (Eisenstein and Williams, 2009) were experimentally observed earlier, thus further validating the model. The model also predicted three novel complex phenotypes, Th1–Th2–iTreg, Th1–Th17–iTreg, and Th1–Th2–Th17–iTreg, for which experimental validation is foreseeable.

### Cytokine Composition Establishes T Cell Phenotypes

**FIGURE 4:**
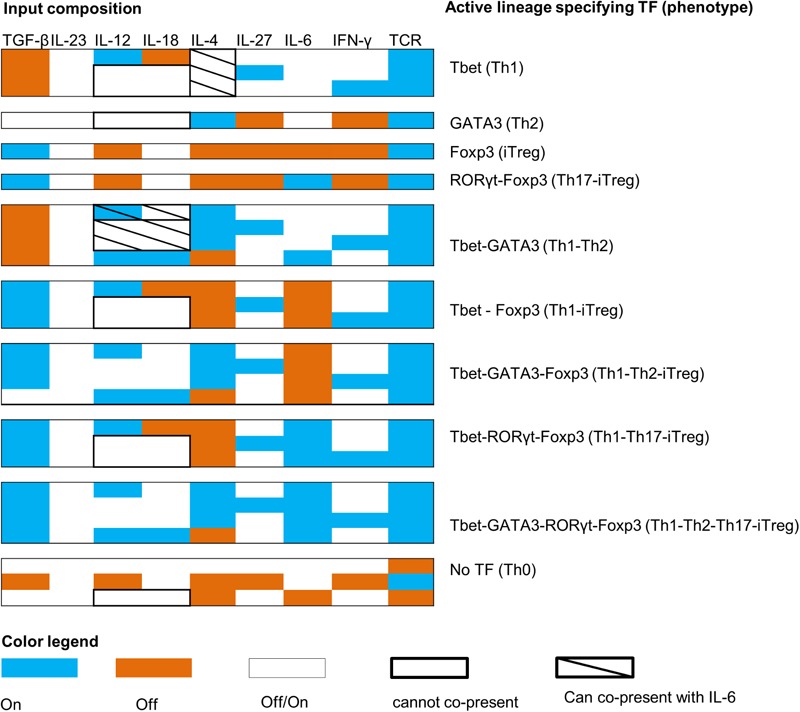
Input compositions for all T cell phenotypes. The color map shows the patterns of input compositions that give rise to each observed phenotype. For example, co-expression of RORγt–Foxp3 needs TCR + IL-6 + TGF-β (+IL-18 and IL-23 can also be active) to be active and IL-12, IFN-γ, IL-27, and IL-4 to be inactive.

**FIGURE 5:**
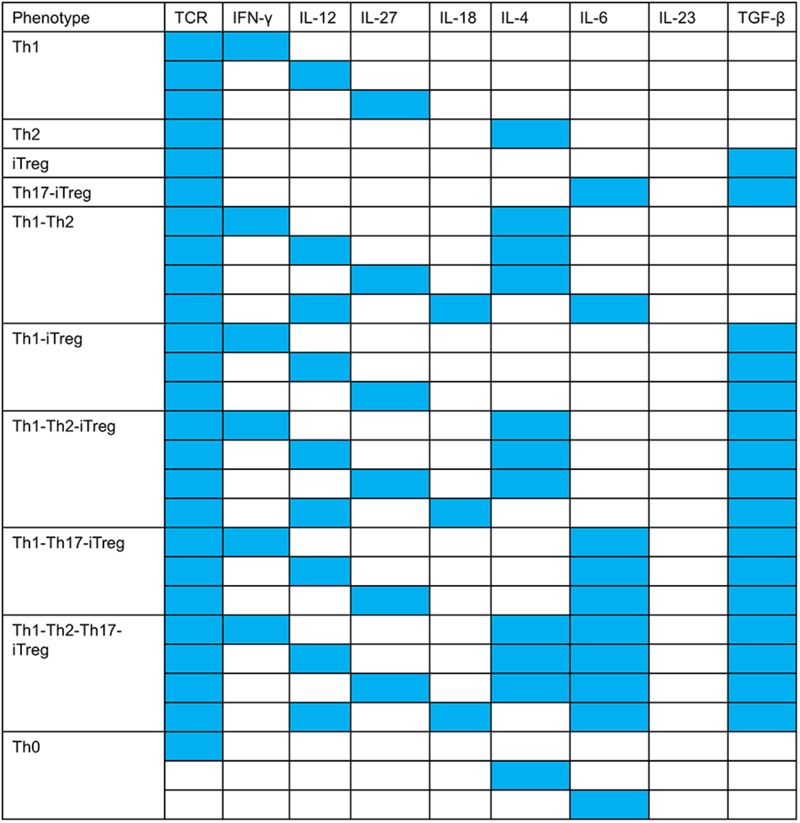
Minimal input compositions required to stimulate T cell phenotypes. All inputs in blue boxes are required to stimulate the corresponding phenotype. For example, the Th1–Th2 phenotype can be stimulated by input composition: TCR ligand + (IFN-γ OR IL-12 OR IL-27) + (IL-4 OR IL-12 + IL-18 + IL-6). Minimum three inputs are required to stimulate the Th1–Th2 phenotype (e.g., TCR ligand + IFN-γ/IL-12/IL-27 + IL-4).

**FIGURE 6:**
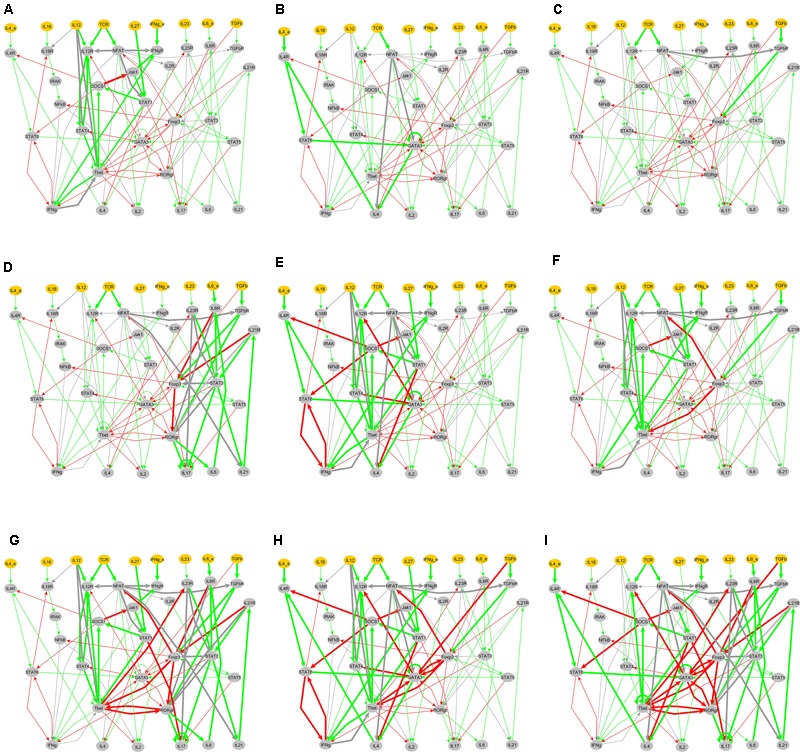
Active signal transduction sub-networks for T cell phenotypes. The active sub-networks under an input composition are mapped on the entire network of T cell differentiation and are marked by bold arrows. Green arrows represent activation and red arrows represents inhibition, and gray arrows represent the condition associated with activation and inhibition. **(A)** Th1 phenotype under input composition: TCR-ligand + IL-12 + IL-27 + IFN-γ, **(B)** Th2 phenotype under input composition: TCR-ligand + IL-4, **(C)** iTreg phenotype under input composition: TCR + TGF-β, **(D)** Th17-iTreg phenotype under input composition: TCR-ligand + IL-6 + TGF-β, **(E)** Th1–Th2 phenotype under input composition: TCR-ligand + IL-4 + IL-12 + IL-27 + IFN-γ, **(F)** Th1–iTreg phenotype stimulated under input composition: TCR-ligand + IL-12 + IL-27 + IFN-γ + TGF-β, **(G)** Th1–Th17–iTreg phenotype stimulated under input composition: TCR-ligand + IL-12 + IL-27 + IFN-γ + IL-6 + TGF-β, **(H)** Th1–Th2–iTreg phenotype stimulated under input composition: TCR-ligand + IL-4 + IL-12 + IL-27 + IFN-γ + TGF-β, **(I)** Th1–Th2–Th17–iTreg phenotype stimulated under input composition: TCR-ligand + IL-4 + IFN-γ + IL-6 + TGF-β.

Once the classification of all the model outputs into biologically relevant phenotypes was carried out, we analyzed the input compositions (environmental conditions) leading to each of the 10 biological phenotypes. This analysis resulted in 27 patterns of input compositions (**Figure 4**). We also identified the minimal input compositions that are needed to stimulate each phenotype (**Figure 5**). Additionally, the signal transduction sub-networks activated for each phenotype, simulated under a representative input composition, are shown in **Figure 6**.

As indicated in the model validation section, we found that the canonical phenotypes (Th0, Th1, Th2, and iTreg) are regulated by one or more cytokines. We also found that all the complex phenotypes can be stimulated by more than one input composition. Strikingly, our modeling effort predicts that in order to induce specific phenotypes, certain cytokines cannot be co-present in a given input composition (**Figures 4, 5**). For example, based on our model, TGF-β should not be present in the input compositions leading to the Th1–Th2 phenotypes, and IL-6 should be absent from the input compositions that lead to iTreg, Th1–iTreg, and Th1 phenotypes. On the other hand, IL-4 can be present in the input composition leading to Th1, but only when co-present with IL-6. IL-4 also needs to be absent in input compositions leading to iTreg, Th17–iTreg, Th1–iTreg, and Th1–Th17–iTreg phenotypes. Finally, IL-12 and IL-18 cannot be co-present in the extracellular environment that stimulates differentiation into Th1, Th2, Th1–iTreg, Th1–Th17–iTreg, and Th0 (in the absence of the TCR ligand) phenotypes.

The previously mentioned heterogeneous and conditional effect of combining IL-12 and IL-18 is also supported and partially explained through experimentally described regulatory mechanisms (Yoshimoto et al., 1998; Nakanishi et al., 2001). Specifically, we observed that combining IL-18 with IL-12 favors co-expression of Tbet, GATA3, and Foxp3. It was previously shown that combining IL-12 and IL-18 can synergistically increase the Tbet-stimulated IFN-γ production in Th1 cells (Tominaga et al., 2000). In another study, it was shown that IL-18, but not IL-12, increases the production of IFN-γ by CD8+ and CD4^+^ T cells in the K14E7 transgenic skin (Gosmann et al., 2014). Further, the combination of IL-12 and IL-18 has been shown to induce the production of IFN-γ in the absence of antigen (Munk et al., 2011). Finally, it has been shown that IL-18 in the absence of IL-12 can stimulate Th2 response (Nakanishi et al., 2001).

**FIGURE 7:**
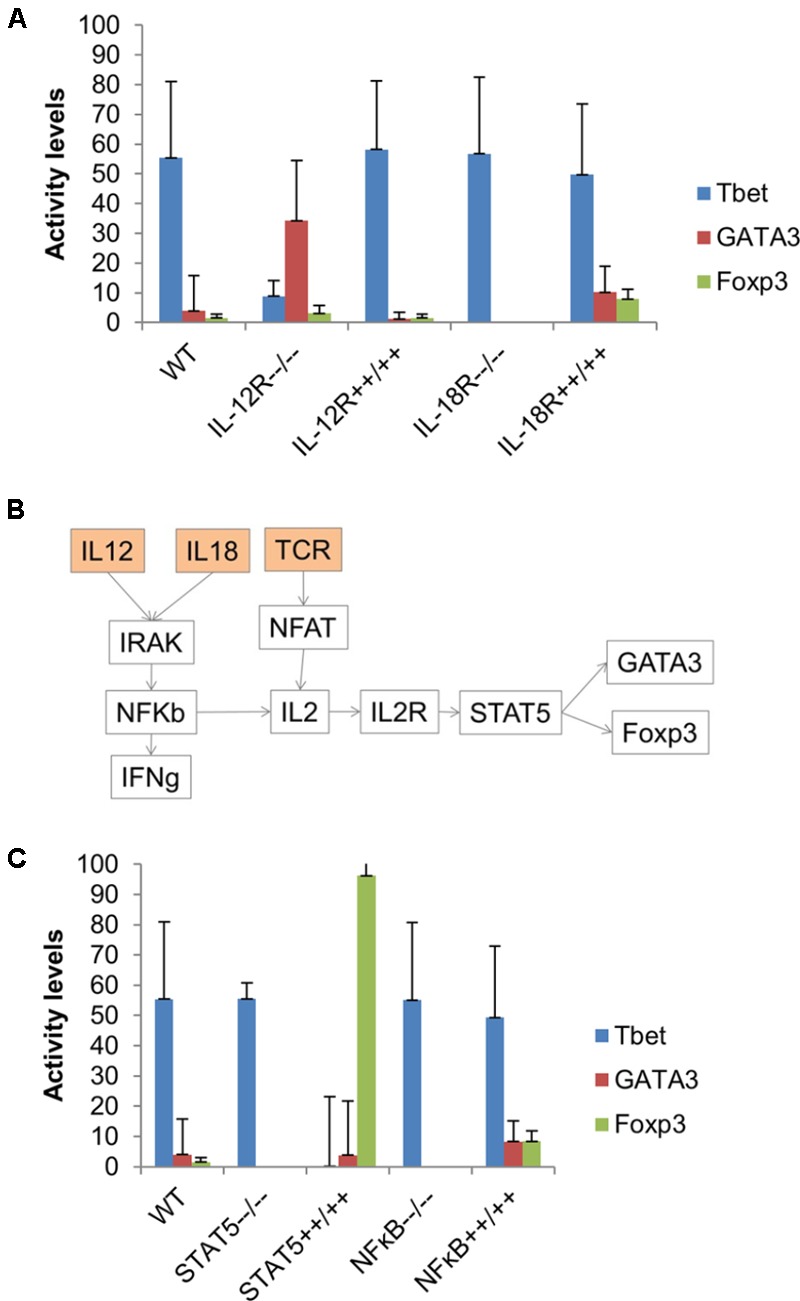
Combination of IL-12 and IL-18 favors the Th1–Th2–iTreg phenotype. **(A)** The model was simulated using input composition (TCR ligand + IL-12 + IL-18) under five conditions, including wild-type, and knock-out and overexpression of both IL-12R and IL-18R. Overexpression of IL-12R favors Th1 phenotype, whereas IL-12R knock-out significantly increased the activity level of GATA3. IL-18R knock-out completely inactivated both GATA3 and Foxp3. **(B)** A subnetwork that might induce GATA3 and Foxp3 in the presence of IL-12 and IL-18, but in the absence of IL-4 and TGF-β. **(C)** Knock-out of STAT5 and NF-κB resulted in complete inactivation of GATA3 and Foxp3, whereas overexpression of STAT5 induced a strong Foxp3 response. Overexpression of NF-κB increased the activity of both GATA3 and Foxp3 by more than twofold.

To further investigate the effect of IL-12 and IL-18 on the Th1–Th2–iTreg phenotype, the model was simulated under the input composition of IL-12, IL-18, and TCR (with all other cytokines inactive). Simulation results suggested the synergistic effect of IL-12 and IL-18 on the activity level of GATA3 and Foxp3. Interestingly, the increased activity of GATA3 and Foxp3 was observed in the absence of external IL-4 and TGF-β (**Figure 7A**), suggesting that the combination of IL-12 and IL-18 (while controlling for the TCR signal) are able to stimulate the Th1–Th2–iTreg phenotype in an IL-4- and TGF-β-independent manner. We also found that the combination of IL-12 and IL-18 is a weaker activator of GATA3 and Foxp3 (**Figure 7A**). This is because the IL-12 can also stimulate Tbet, which in turn suppresses the GATA3 and Foxp3. Results obtained from the simulated IL-12R knock-out suggested an eightfold increase in the activity of GATA3, whereas the overexpression of IL-12R slightly decreased the activity levels of GATA3 and Tbet. Knock-out of IL-18R resulted in a complete inactivation of GATA3 and Foxp3, whereas the overexpression of IL-18R resulted in a greater than twofold increase in the activity levels of GATA3 and Foxp3. These results indicate that the knock-out of IL-12R favors Th2 phenotype, whereas the knock-out of IL-18R favors Th1 phenotype under Th1–Th2–iTreg stimulating environmental conditions.

The differentiation to Th2 was previously observed in airway epithelia in the presence of IL-18, but not IL-4 (Murai et al., 2012). The IL-4-independent Th2 stimulation possibly occurs through the STAT5-mediated GATA3 activation (Yamane et al., 2005; Paul, 2010). The IL-18R1 signaling was also found to promote Foxp3+ iTreg cell function within colonic lamina propria (Harrison et al., 2015). To better understand the mechanism of how the IL-18 and IL-12 can stimulate GATA3 and Foxp3, we further analyzed the network structure of the model. We found that IL-12 and IL-18 can possibly induce the production of IL-2, which stimulates GATA3 and Foxp3 in STAT5-dependent pathways (**Figure 7B**). The knock-out simulation of NF-κB or STAT5 resulted in complete inactivation of GATA3 and Foxp3. On the other hand, the overexpression of STAT5 increased the mean activity level of Foxp3 by 62-fold, while no change in activity levels of GATA3 was observed. The simulated over-expression of NF-κB had shown 5.4-fold and twofold increase in the activity levels of Foxp3 and GATA3, respectively. These results predict the role of IL-12 and IL-18 in stimulation of the Th1–Th2–iTreg phenotype in an NF-κB- and STAT5-dependent manner (**Figure 7C**). Furthermore, our simulation results suggest that a combination of IL-18 and IL-12 can stimulate Tbet, GATA3, and Foxp3; however, the activity levels of GATA3 and Foxp3 were lower than that of Tbet (**Figure 7A**). Additionally, we have found that IL-12 and IL-18 combination in the presence of IL-6 can stimulate the Th1–Th2 phenotype (Supplementary Figure 1 in Supplementary Datasheet 3).

Altogether, we have identified input composition patterns that include the minimum combinations of cytokines required to stimulate a particular T cell phenotype, as well as complete pattern of cytokines that can be co-present to stimulate a given phenotype (Supplementary Table 3). Our results also predict the relevance of IL-12 and IL-18 in regulating the Th1–Th2–iTreg phenotype. Finally, we predicted an alternative pathway that can stimulate GATA3 and Foxp3 in an IL-4 and TGF-β-independent manner.

### Cytokine Dosage Determines the Balance Between Complex T Cell Phenotypes

In the previous section, various input compositions that lead to different canonical and complex phenotypes were characterized. The logical question that we raise now is: How is the balance of each T cell subtype within a complex phenotype controlled?

As indicated in the “Introduction” section, several reports suggest that the balance between Th17 and iTreg is regulated by the dosage of IL-6 (Kimura and Kishimoto, 2010; Omenetti and Pizarro, 2015). To explore how the input dosages within each composition affect the complex phenotypes, we analyzed the model under various activity levels of cytokines and the TCR ligand under the complete set of input compositions.

We used the representative input compositions for each identified phenotype as described in **Figures 4, 5**. Specifically, we used two types of representative input compositions from each row in **Figure 4**. The two types include, one with the maximum number of inputs that can be simultaneously present to stimulate a specific T cell phenotype, and a second type that is represented by input compositions consisting of the minimal number of inputs required to stimulate the identified phenotypes.

**FIGURE 8:**
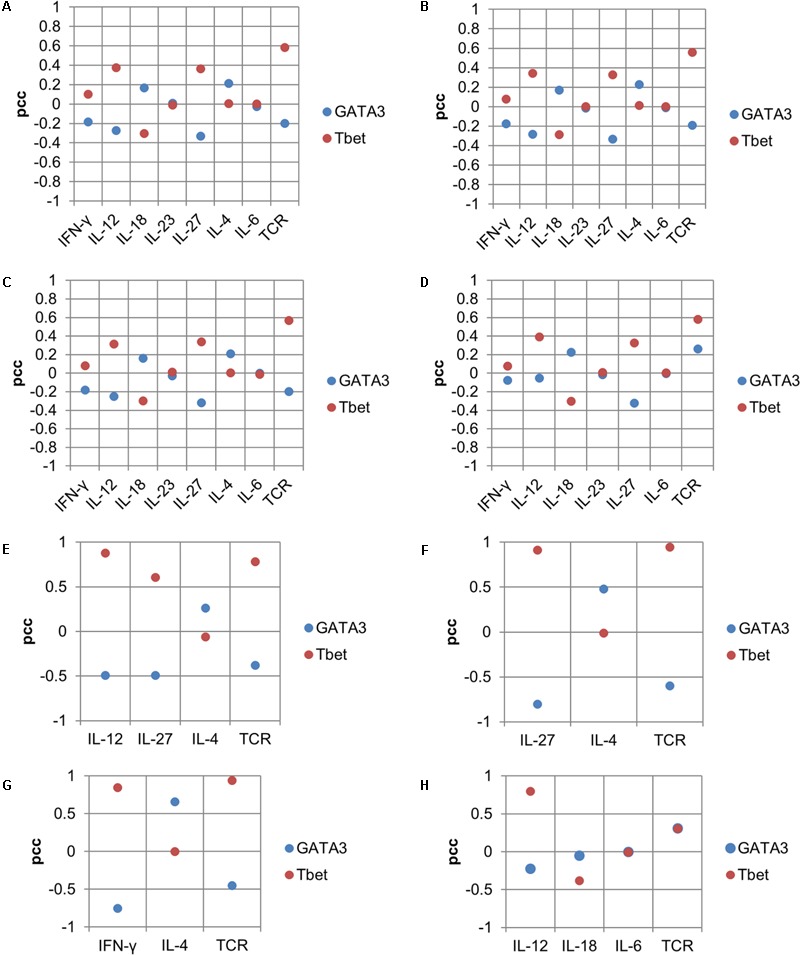
Sensitivity analysis showing the input effect on lineage-specifying TFs for the Th1–Th2 phenotype. Panels **(A–D)** are based on simulations using maximal input compositions. Panels **(E–H)** are based on minimal input compositions. PCC as a measure of association between inputs (cytokines and TCR) and lineage-specifying TFs is shown on *Y*-axes and input composition (cytokines and TCR) is shown on *X*-axes.

Sensitivity analysis of the model was performed to describe the effect of each input in its composition on the TF(s) for the corresponding complex phenotype (**Figure 4**). The analysis predicted individual inputs that are important for regulating the balance among lineage-specifying TFs. For example, for the Th1–Th2 phenotype, when stimulated with a maximum of eight inputs, the sensitivity analysis suggested that IL-27, IFN-γ, and IL-12 are negatively correlated with GATA3 (**Figures 8A–C**). The TCR signal is negatively correlated with GATA3 [partial correlation coefficient (PCC) range = -0.18 to -0.19] under three input compositions (**Figures 8A–C**). Interestingly, a positive correlation between the TCR ligand and GATA3 was observed when the Th1–Th2 phenotype was stimulated in the absence of IL-4 (and in the presence of IL-12, IL-18, and IL-6) (**Figure 8D**). On the other hand, the IL-18 had a moderate negative correlation with Tbet (PCC range = -0.28 to -0.29) under all tested input compositions (**Figures 8A–D**). The IL-4 had a very low correlation with Tbet (PCC range = 0.005–0.01) under all tested input compositions (**Figures 8A–D**).

Next, in the case of the Th1–Th2 phenotype stimulated under minimal input compositions, higher correlations between the inputs and TFs were observed compared to the correlations observed with maximal input compositions (**Figures 8E–H**). In the case of the input composition “TCR + IL-12 + IL-4,” IL-12 had a strong negative correlation (PCC = -0.68) with GATA3, and a strong positive correlation with Tbet (PCC = 0.7). In this case, the TCR ligand had a moderate negative and positive correlation with GATA3 (PCC = -0.25) and Tbet (PCC = 0.22), respectively. In the case when Th1–Th2 phenotype was stimulated under input composition “TCR + IL-12 + IL-18 + IL-6,” the TCR ligand was positively correlated with both GATA3 (PCC = 0.30) and Tbet (PCC = 0.30). A strong positive correlation was observed between IL-4 and GATA3 (PCC = 0.65) under the input composition “TCR + IFN-γ + IL-4.” In the Th1–Th2 complex phenotype, we observed that the TCR ligand is negatively correlated with GATA3. The negative effect of a strong TCR ligand signal on GATA3 is in agreement with the earlier studies suggesting that a strong TCR signal can promote a strong Th1 response, whereas a weaker signal favors the Th2 response (van Panhuys et al., 2014). The sensitivity analysis results for all other mixed phenotypes are provided in Supplementary Table 4.

In summary, the sensitivity analysis of our model predicts “driver” inputs. Furthermore, it characterizes the strength and direction (positive or negative) of the effect inputs can have on the regulation of the balance of each T cell subtype within the complex phenotypes. The strength of association between the inputs and TFs varied based on the number of inputs in the input compositions.

### Determining the Optimal Input Dosage Regulating the Balance Between Complex Phenotypes

**FIGURE 9:**
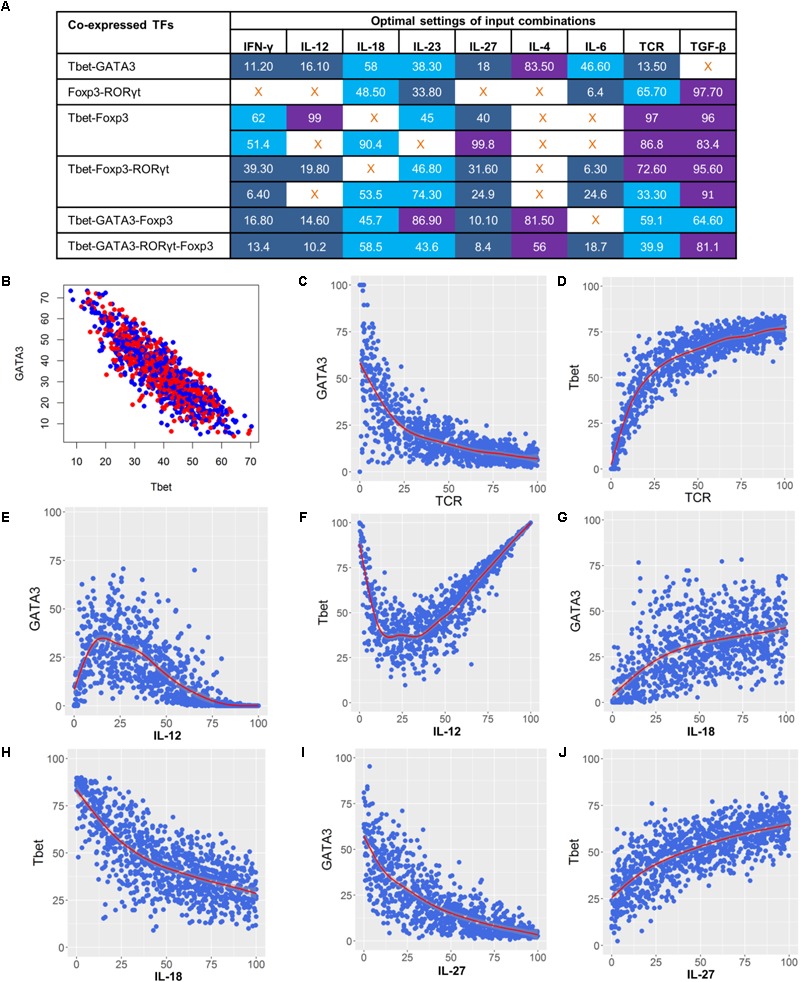
Dose-dependent regulation of complex phenotypes co-expressing lineage-specifying TFs. **(A)** Optimal settings using maximal input composition that stimulate co-expression of lineage-specifying TFs. Shown are median activity levels of inputs that result in a balanced activity level of both TFs. Activity levels ranging from 0 to 40 (blue) were considered as low, 40 to 80 were medium (light blue), and 80 to 100 were high (purple). **(B)** Activity levels of GATA3 and Tbet when simulated under optimal input settings that stimulate the Th1–Th2 phenotype. Median activity levels from panel A were used for the simulation. **(C–J)** Dose-response curves illustrating the effect of TCR, IL-12, IL-18, and IL-27 on GATA3 and Tbet.

In the previous sections, the predicted complex T cell phenotypes, input compositions, as well as the potential dosage effect each input can have on the phenotype, were discussed. Next, we examined the specific activity levels of the input compositions required to control each specific T cell phenotype. The model was simulated under 10,000 randomly generated environmental conditions within the context of each relevant input composition. Results from these simulations provided us with specific input activity levels that have a low coefficient of variance (CV) in activity levels of co-expressed lineage-specifying TFs. Specifically, we investigated and characterized the activity levels for each input composition that will drive a complex T cell phenotype to each of the T cell subtypes or a balanced mixed phenotype by maximizing the activity levels of the respective TFs. For example, to achieve a balanced Th1–Th2 phenotype that has similar activity levels to that of GATA3 and Tbet, we characterized the optimal activity levels for each input in the Th1–Th2-leading input compositions. This predicted optimal input composition includes the low activity of the TCR ligand, IFN-γ, IL-12, and IL-27, medium activity of IL-18 and IL-6, and high activity of IL-4. The activity of IL-23 can vary from low to high, whereas TGF-β should be inactive (**Figure 9A**).

To illustrate the effect of using optimal activity levels, we stimulated the Th1–Th2 phenotype by using a median value of optimal activity level for each input. As expected, the simulation results show similar activity levels of Tbet and GATA3 (**Figure 9B**). To further investigate the effect of dominant inputs (identified from the sensitivity analysis) on the Tbet–GATA3 combination, we performed dose-response analysis by varying the dominant cytokines while fixing the other inputs to median activity levels (**Figure 9A**). As expected, our results (**Figures 9C–J**) suggest that the increased signal strength of TCR ligand or increased activity of IL-12 and IL-27 can drive the Th1–Th2 phenotype toward Th1 by increasing the activity of Tbet and decreasing the activity of GATA3. In contrast, the increased activity of IL-18 can drive Th1–Th2 phenotypes toward Th2.

## Discussion

In this study, we sought to investigate the cellular phenotypes as a result of CD4^+^ T cell differentiation under diverse environmental conditions and understand how the balance between complex phenotypes is regulated. To achieve this, by manually curating literature data, we constructed a mechanistic computational (logical) model of signal transduction that regulates the differentiation of naive T cells into Th1, Th2, Th17, and iTreg cells. The components (i.e., proteins and genes) in a logical model can have binary (0 or 1) states at any time *t*. The state of the network evolves stepwise based on the logical rules defined for each model component (Helikar and Rogers, 2009; Helikar et al., 2012a,b, 2013; Naldi et al., 2015; Abou-Jaoudé et al., 2016; Barberis and Verbruggen, 2017; Linke et al., 2017).

We systematically characterized the model’s dynamics in the context of activity of lineage-specifying TFs under 511 input compositions consisting of eight cytokines and a TCR signal. In addition to the dynamics representing the classical Th0, Th1, and Th2 phenotypes, we found several complex (mixed) phenotypes (dynamics with more than one lineage-specific TFs), including Th1–Th2, Th1–iTreg, Th17–iTreg, Th1–Th2–iTreg, Th1–Th17–iTreg, and Th1–Th2–Th17–iTreg. Our results are in agreement with recent studies that reported hybrid T cell phenotypes *in vitro* and *in vivo* (Zhou et al., 2008; Peine et al., 2013). Stable complex Th1–Th2 phenotypes parallel to the classical Th2 phenotypes were observed *in vivo* upon infection mediated by parasites *Schistosoma mansoni* and *Heligmosomoides polygyrus* (Peine et al., 2013), as well as by the threadworm *Strongyloides stercoralis* (Bock et al., 2017). Moreover, Th1–iTreg intermediate phenotypes were observed during Th1 polarizing infections (Koch et al., 2009; Oldenhove et al., 2009; Evans and Jenner, 2013). In a recent system level study, a continuum of T cell differentiation states with stable co-expressed lineage-specific TFs has been observed when stimulated under different combinations of six cytokines (Eizenberg-Magar et al., 2017).

Interestingly, we did not observe a canonical Th17 (RORγt-only) phenotype. Instead, our model predicts the existence of a mixed Th17–iTreg phenotype. This result can be partially explained by the fact that both Th17 and iTreg share a common mechanism by cytokine TGF-β, and the differentiation of naive T cells into iTreg or Th17 depends on the cytokine-driven (TGF-β and IL-6) balance of lineage-specifying TFs Foxp3 and RORγt (Omenetti and Pizarro, 2015). In addition, it is known that the Th17/Treg balance is critical to maintain immune tolerance. The imbalance of Th17/Treg has been observed in the peripheral blood of cervical cancer patients (Chen et al., 2013), non-small cell lung cancer patients (Duan et al., 2015), and in patients with chronic low back pain (Luchting et al., 2014). Thus, the complex Th17–iTreg phenotype might play an important role in maintaining Th17/Treg homeostasis. Such complex RORγt–Foxp3 co-expressing T cells were observed in an autoimmune diabetes model (Ichiyama et al., 2008; Tartar et al., 2010), in the lamina propria (Zhou et al., 2008), in the peripheral blood and tonsils (Voo et al., 2009), and in the large intestine (Ohnmacht et al., 2015; Fang and Zhu, 2017). It is also possible that the lack of Th17-only phenotype is due to the incomplete nature of the model. However, it suggests that additional experimental validation may be required to better understand the relationship and mechanism of switching between iTreg and Th17 phenotypes.

We also predicted novel phenotypes that have the potential to have three active TFs (Tbet–GATA3–Foxp3, Tbet–RORγt–Foxp3), as well as one with all four TFs (Tbet–GATA3–RORγt–Foxp3). In partial support of our prediction, basal levels of Tbet and GATA3 have been observed in iTreg cells (Yu et al., 2015). While not yet shown experimentally, the Th1–Th17–iTreg phenotype was also predicted by a similar modeling approach (Naldi et al., 2010).

By analyzing all possible inputs combinations, we obtained the minimal and maximal input compositions for each identified phenotype. The minimal composition includes a minimum number of inputs that can stimulate a phenotype. On the other hand, the maximal composition includes a maximum number of inputs that can be simultaneously active to result in the same phenotype. In this analysis, we found that in order to stimulate Th1, Th2, Th1–iTreg, Th1–iTreg, Th1–Th17–iTreg, and Th0 phenotypes, IL-12 and IL-18 cannot be combined in the environment. We observed that the combination of IL-12 and IL-18 leads to the stimulation of GATA3 and Foxp3 even in the absence of IL-4 and TGF-β via a NF-κB-dependent pathway. We predicted that a combination of IL-18 and IL-12 could result in a Th1–Th2–iTreg complex phenotype. Analysis of the model’s network structure suggests a potential mechanism that is dependent on NF-κB and STAT5 (**Figure 7B**). Previous studies suggest that IL-18 has a context-specific functional heterogeneity and can induce both Th1 and Th2 T cell phenotypes. The combination of IL-12 and IL-18 has been shown to have a synergistic effect on IFN-γ production that stimulates the Th1 phenotype (Tominaga et al., 2000; Munk et al., 2011). It has also been found that IL-18 alone (without IL-12) can stimulate the Th2 phenotype (Nakanishi et al., 2001). In a study on airway epithelial cells in response to *Alternaria*, it was found that secreted IL-18 has the capacity to stimulate the Th2 phenotype (Murai et al., 2012). Since IL-12 can up-regulate IL-18R expression, it might be possible that the combination of IL-12 and IL-18 may regulate the Th1, Th2, Th1–Th2, and Th1–Th2–iTreg phenotypes in a dose-dependent manner.

Next, the sensitivity analysis of the model suggested that the dosage of the individual inputs regulates the balance within the different complex T cell phenotypes. We investigated the dosage effect by using both minimum and maximum number of inputs under varying activity levels. For example, our results suggest that the dynamics of the complex Th1–Th2 phenotype depend on the combination and dosage of IFN-γ, IL-12, IL-27, IL-18, IL-4, and the TCR ligand. The increased activity levels of the cytokines IFN-γ, IL-12, IL-27, and TCR ligand drive the phenotype toward Th1, whereas the IL-18 or IL-4 drive the Th2 phenotype. The IL-23 and IL-6 have no correlation with either Tbet or GATA3. Under both maximal and minimal input compositions, the IL-4 had low to no correlation with Tbet. On the other hand, the IL-18 was positively correlated with GATA3 and negatively correlated with Tbet. Thus, we predicted that IL-18 may have a dominant role over IL-4 to favor Th2 phenotype under the Th1–Th2 stimulating environmental conditions.

Next, we identified the activity levels of the inputs required to regulate the complex T cell phenotypes. Our results suggest a range of activity levels required to obtain a specific phenotype under minimal and maximal input compositions. For example, a high amount of IL-4 or IL-18 and a low amount of IFN-γ, IL-12, IL-27, and TCR ligand are required to stimulate the Th1–Th2 phenotype under maximal input composition. Low activity of GATA3 under higher TCR ligand activity is indeed in agreement with the literature where it has been shown that a strong TCR signal represses GATA3 (Aguado et al., 2002; Yamane et al., 2005; Paul, 2010; Altin et al., 2011; Yamane and Paul, 2012). Interestingly, our results showed an increase as well as a decrease in the activity levels of GATA3 depending on the activity levels of IL-12. This can be achieved as a result of IL-12 up-regulating IL-18R, which induces NF-κB-mediated GATA3 activation. On the other hand, a higher activity of IL-12 results in a strong Tbet activation, which in turn suppresses GATA3. Although the predicted activity levels are dimensionless and semi-quantitative, they provide a starting point for calibrations against ligand concentrations in specific experimental research protocols.

In summary, results provided in this study can provide a platform to generate and design testable hypotheses in the context of T cell differentiation in response to various combinations and dosage of environmental signals. Furthermore, the presented results and the mechanistic model can be used as tools to further investigate the specific pathway mechanisms that govern each complex phenotype. Input availability and relative dosage at which inputs generate a productive signaling cascade necessarily result in a variable timing of an immune response. Specifically, we and others propose that dosage- and timing-dependent impact of inputs, such as ILs, may impact the T cell differentiation (Barberis et al., 2018; Martinez-Sanchez et al., 2018). This may be investigated by employing experimental methodologies that we have recently envisioned (Barberis and Verbruggen, 2017). Furthermore, crosstalk between ILs and signaling cascades, such as the one governing the cell cycle, may impinge on a timely T cell-mediated protective response (Barberis et al., 2018). These aspects are the focus of our current research efforts. Together with new model-based predictions, improving the understanding of the detailed mechanisms underlying T cell differentiation, can be helpful to design strategies for immunotherapy against pathogens and various diseases of the immune system.

## Materials and Methods

### Model Construction

The computational model is a mechanistic, logic-based model of signal transduction processes known to regulate CD4^+^ T cell differentiation into Th1, Th2, Th17, and iTreg cells. Each component of the model can assume an active (1) or inactive (0) state at any time *t*. The activity state of the model’s internal components is determined by the regulatory mechanisms of other directly interacting components. These regulatory mechanisms are described with Boolean functions (Samaga and Klamt, 2013; Albert and Thakar, 2014; Le Novère, 2015; Naldi et al., 2015; Abou-Jaoudé et al., 2016; Linke et al., 2017).

The new signal transduction model was constructed manually by curating published regulatory mechanisms of each signal transduction component. Each of the 38 components in the model corresponds to a signaling molecule (mainly proteins). The model also contains nine external components that represent the extracellular environment, consisting of eight cytokines (IFN-γ, TGF-β, IL-4, IL-6, IL-12, IL-18, IL-23, and IL-27) and a generic TCR ligand. The final model consists of 38 components (29 internal and 9 external) connected with 96 interactions. The model is fully annotated with published evidence for each component and interaction to ensure transparency and reproducibility. The model is available via the web-based modeling and analysis platform Cell Collective (Helikar et al., 2012b, 2013), accessible at https://www.cellcollective.org (under Published Models) where it can be simulated as well as downloaded (and other logical models published by the community) in several file formats (such as SBML-qual, text file of logical functions, and truth tables).

### State Space Analysis

The logical model herein is a *Probabilistic Boolean Control Network (PBCN)* (Todd and Helikar, 2012), whereby each external input (components that are not regulated by other model components) is activated by a user-defined probability of activation (ranging from 0 to 100%). The activity levels of the external inputs and the logical rules associated with each internal node allow the system to update stochastically in time. As such, a *PBCN* is a reducible Markov chain (Tijms, 2003). We used ergodic sets (recurrent communicating classes of the corresponding Markov chain) as a model of stable cell states that represent the phenotype of a differentiated T cell. Ergodic sets are a collection of states in state space such that once the system evolves to one of these states it will remain in this set of states. In this way, the ergodic sets are the stochastic equivalents to attractors in purely Boolean networks (Ribeiro and Kauffman, 2007).

From each initial condition, the system will arrive in one of a (possibly) different collection of ergodic sets. In order to find all the ergodic sets, one would need to let the system evolve from every possible initial condition. Given the large number of possible initial conditions (2^29^), this is computationally infeasible. Thus, we found those ergodic sets that can be reached from the initial state where all internal components are inactive. This represents our goal, i.e., to identify cell phenotypes that are the result of differentiation from naive T cells (i.e., all model components are inactive). Once an ergodic set was identified it was treated as an *irreducible* Markov chain and thus has an associated limiting distribution. Activities of the internal components are interpreted by approximating the limiting distribution of the Markov chain via simulations in Cell Collective. This means that each internal component has a unit less activity level corresponding to the probability that it is active in the limiting distribution of the Markov chain.

### Identification of Ergodic Sets

The extracellular environment (external input components) in the presented model consists of nine stimuli — eight cytokines and a generic TCR ligand. A given extracellular environment is described according to those stimuli that are *off* (no activity) and those that are *on* (some level of activity). Thus, there are 2^9^ = 512 possible *off*/*on* configurations for the extracellular environment (input compositions). The ergodic sets that are reachable from the naive state (where all components are inactive) depend only on this *off*/*on* description and not on the activity level of the non-off cytokines. We were able to identify the corresponding reachable ergodic sets for 508 of these input compositions. The only extracellular environments that are yet unknown are the three where all stimuli are *on* except for TGF-β, or IL-23, or IL-4. The ergodic sets were identified in two steps. In the first step, Tarjan’s algorithm (Tarjan, 1972) was used to identify communicating classes of states. In the second step, these classes were directly tested to determine if they were closed. The ergodic sets (other than the fixed points) ranged in size from the smallest, with two states, to the largest with 594,962 states. These ergodic sets correspond to the “outputs” in **Figures 3A,B**. Each state in an ergodic set specifies the state of the internal network. In order to classify an ergodic set, for each internal component we computed the percentage of states in which the component was active. For example, the ergodic set that was identified when the TCR ligand and IL-4 are *off* while all other external stimuli are *on*, was found to have 64 states. Each of IL-18R, IL-4R, IRAK, NF-κB, and STAT6 are *on* in 50% of states, though not the same 50% of states. All other internal components were *off* in all of the 64 states. In this case, as no lineage-specific TFs are expressed at any level, it is classified as a Th0 phenotype.

The computations to find the ergodic sets were implemented in PERL and were run on an 82-node Linux cluster. Most computations of the ergodic sets required around 10–20 gigabytes of RAM and took from hours to days for the Tarjan’s algorithm to find an ergodic set. (Some required much more). In general, given an initial condition and *off*/*on* input composition, several ergodic sets could be reached. We found that out of the 512 possible input compositions, 502 compositions lead to a unique ergodic set and 6 of them lead to exactly two ergodic sets. There were three input compositions that led to one ergodic set, but for which the algorithm had not finished the complete search even after 7 days of calculations. Thus, for these three input compositions, there could be reachable ergodic sets that we did not identify. One input composition, in which all external inputs are active, ran for 7 days without finding any ergodic sets (this is the only input composition for which we have no ergodic set). As we got inconclusive results from the aforementioned incomplete analyses, the corresponding four input compositions were excluded from any reported results.

### Model Simulations in Cell Collective

Model simulations were performed in the web-based modeling platform, Cell Collective^2^. Although the model is built by using discrete mathematics, the output activity levels of individual components can be represented as semi-continuous values ranging from 0 to 100% as previously described in Helikar et al. (2008) and Helikar and Rogers (2009). Each simulation was conducted using synchronous updates, and consisted of 5,000 steps, where the activity level of the measured output component was calculated as the fraction of ones (active states) over the last 500 iterations that describe the model’s steady behavior (Helikar et al., 2008; Helikar and Rogers, 2009). The activity levels (dosage) of external components is unit-less and defined as a per-cent chance (probability ^∗^ 100) of the component being active in a given time *t*. Depending on the desired experiment, the activity levels of external components can be set by the user to specific values, or they can be set to ranges from which values during each simulation are selected randomly (e.g., to simulate dose-response experiments).

Once the ergodic sets were identified, expressions of the internal components and their dependencies on the dosages of the external cytokines and the TCR ligand were investigated via the Cell Collective (Helikar et al., 2012b).

For each ergodic set, we chose one of its states as an initial condition and then simulated the model with the corresponding extracellular conditions via the Cell Collective. For each of the active input cytokines, the activity levels varied between 1 and 99%. Further details of the use of the Cell Collective are specific to the types of analysis as described below.

### Sensitivity Analysis

The model was simulated in Cell Collective, whereby the activity levels of the inputs for each composition varied. By using the model-generated simulation data under 10,000 randomly generated environmental conditions, the association between inputs (cytokines and TCR ligand) and outputs (lineage-specifying TFs) was determined by probabilistic global sensitivity analysis based on PCC using the “*sensitivity*” package in R (R Development Core Team, 2011; Pujol et al., 2017). The PCC measures the strength of association between the output and input parameters after removing the linear effect of other input parameters (Marino et al., 2008; Pujol et al., 2017). The PCC between input and output is the correlation coefficients between residuals (x_j_ - 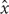
_j_) and (y -ŷ), where *x_j_* and *y* are input and output, respectively, and 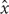
_j_ ŷ are linear regression models [shown in Equation (1)] (Marino et al., 2008).

(1) $${\hat{x}}_{j}=c_{0}+\sum_{\begin{array}{l} p\;=\;1\\ p\;\neq \;j \end{array}}^{k}c_{p}x_{p}\;\mathrm{and}\;\;\hat{y}=b_{0}+\sum_{\begin{array}{l} p=1\\ p\;\neq \;j \end{array}}^{k}b_{p}x_{p}.$$

### Optimal Settings Analysis

Once again, the model was simulated using 10,000 randomly generated environmental conditions for each input composition that can stimulate a complex phenotype. We sought to identify the environmental conditions wherein multiple lineage-specifying TFs can have balanced activity levels. First, we used the CV [Equation (2)] between TFs to measure variability. Further, we selected simulation results under which the lowest variability between TFs was observed. We selected corresponding environmental conditions that had lowest CV among TFs. Next, we selected the top 10 environmental conditions based on the outputs that have the highest activity levels of TFs. Thus, we considered both the balance of activity levels as wells as the quantity of co-expressed TFs. Finally, we defined ranges of activity levels of inputs from the selected environmental conditions. Further, for Th1–Th2, we simulated the effect of dominant inputs by individually varying IL-12, IL-18, IL-27, and the TCR ligand and using median activity levels from identified optimal activity levels for other inputs. We used R-scripts to determine the optimal activity levels from simulation data obtained via Cell Collective (Helikar et al., 2012b). The effect of dominant inputs on TFs in a complex phenotype was shown using the Generalized Additive Model (GAM) fitted scatter plots generated using “ggplot2” package in R.

(2) $$\%\mathrm{CV}\;=\;\frac{\mathrm{Standard}\;\mathrm{deviation}}{\mathrm{mean}}\;\times \;100.$$

## Author Contributions

BP, RT, DB, MB, and TH designed the research. BP, RT, AM, and DB performed the research. BP, MB, and TH analyzed the data. BP, RT, MB, and TH wrote the paper.

## Conflict of Interest Statement

TH has served as a scientific advisor and/or consultant to Discovery Collective. The remaining authors declare that the research was conducted in the absence of any commercial or financial relationships that could be construed as a potential conflict of interest.

## Footnotes

1 https://www.cellcollective.org

2 https://www.cellcollective.org
