# Supplementary material for: A Mechanistic Computational Model Reveals That Plasticity of CD4+ T Cell Differentiation Is a Function of Cytokine Composition and Dosage
Source: Front Physiol. 2018 Aug 2;9:878. doi: 10.3389/fphys.2018.00878 (PMC6083813; doi:10.3389/fphys.2018.00878)
Supplement: Supplementary file 1 [file Table_1.DOCX]

| **Phenotypes** | **Input cytokine combinations** | **Expected behaviors** | **Reproduced behaviors** | **References** |
| --- | --- | --- | --- | --- |
| Th1 | TCR + IL-12/IFN-γ/IL-27 | Tbet, IFN-γ | Tbet, IFN-γ | (Luckheeram et al., 2012; Zhou et al., 2009; Zhu et al., 2010) |
| Th2 | TCR + IL-4 | GATA3, IL-4 | GATA3, IL-4 | (Luckheeram et al., 2012; Zhou et al., 2009; Zhu et al., 2010) |
| Th17 | TCR + IL-6 (high) + TGF-β | RORγt, IL-17 | RORγt, IL-17 | (Luckheeram et al., 2012; Zhou et al., 2009; Zhu et al., 2010) |
| iTreg | TCR + TGF-β | Foxp3 | Foxp3 | (Luckheeram et al., 2012; Zhou et al., 2009; Zhu et al., 2010) |

**Supplementary Table 1: Classical T-cell differentiation behaviors reproduced by model**

**References:**

Luckheeram, R., Zhou, R., Verma, A., and Xia, B. (2012). CD4+T Cells: Differentiation and Functions. *Clin. Dev. Immunol.* 2012, 1–12. doi:10.1155/2012/925135.

Zhou, L., Chong, M. M. W., and Littman, D. R. (2009). Plasticity of CD4+ T Cell Lineage Differentiation. *Immunity* 30, 646–655. doi:10.1016/j.immuni.2009.05.001.

Zhu, J., Yamane, H., Paul, W. E., and J, Z. J. (2010). Differentiation of effector CD4 T cell populations (*). *Annu. Rev. Immunol.* 28, 445–489. doi:10.1146/annurev-immunol-030409-101212.
