# Supplementary material for: A Mechanistic Computational Model Reveals That Plasticity of CD4+ T Cell Differentiation Is a Function of Cytokine Composition and Dosage
Source: Front Physiol. 2018 Aug 2;9:878. doi: 10.3389/fphys.2018.00878 (PMC6083813; doi:10.3389/fphys.2018.00878)
Supplement: Supplementary file 3 [file Table_3.DOCX]

**Supplementary Table 3: Maximal and minimal input compositions to stimulate T cell phenotypes.**

| **Phenotype** | **Co-expressed TFs** | **Maximum input composition** | **Minimum input composition** |
| --- | --- | --- | --- |
| Th1 | Tbet | TCR + IL-18+IL-27+IFN-γ+IL-4+IL-6+IL-23 | TCR+IL-12/IL-27/IFN-γ |
| Th2 | GATA3 | TCR+ TGF-β + IL-23 + IL-18 (or IL12) + IL-4+IL-6 | TCR + IL-4 |
| iTreg | Foxp3 | TCR + TGF-β + IL-23 + IL-18 | TCR + TGF-β |
| Th17-iTreg | Foxp3-RORγt | TCR + TGF-β + IL-6 + IL-18 + IL-23 | TCR + TGF-β + IL-6 |
| Th1-Th2 | Tbet-GATA3 | TCR + IFN-γ + IL-12 + IL-18 + IL-23 + IL-27 + IL-4 + IL-6 | TCR + IFN-γ/IL-12/IL-27 + IL-4  TCR + IL-12 + IL-18 |
| Th1-iTreg | Tbet-Foxp3 | TCR + IFN-γ + IL-12 + IL-23 + IL-27 + TGF-β | TCR + IFN-γ/IL-12/ IL-27 + TGF-β |
|  |  | TCR + IFN-γ + IL-18 +IL-23 + IL-27 + TGF-β | TCR + IFN-γ/IL-12/ IL-27 + TGF-β |
| Th1-Th17-iTreg | Tbet-Foxp3-RORγt | TCR + IFN-γ + IL-12 + IL-23 + IL-27 + IL-6 + TGF-β | TCR+  IFN-γ/IL-12/ IL-27 + IL-6 + TGF-β |
|  |  | TCR + IFN-γ +  IL-18 + IL-23 + IL-27 + TGF-β | TCR+  IFN-γ/IL-12/ IL-27 + IL-6 + TGF-β |
| Th1-Th2-iTreg | Tbet-GATA3-Foxp3 | TCR + IFN-γ + IL-12 + IL-18 + IL-23 + IL-27 + IL-4 + TGF-β | TCR + IFNγ / IL12 / IL27 + IL4 + TGFβ |
| Th1-Th2-Th17-iTreg | Tbet-GATA3-RORγt-Foxp3 | TCR + IFN-γ + IL-12 + IL-18 + IL-23 + IL-27 + IL-4 + IL-6 + TGF-β | TCR + IFN-γ / IL-12 /IL-27 + IL-18 + IL-23 + IL-4 + IL-6 + TGF-β |
