# Supplementary material for: A Mechanistic Computational Model Reveals That Plasticity of CD4+ T Cell Differentiation Is a Function of Cytokine Composition and Dosage
Source: Front Physiol. 2018 Aug 2;9:878. doi: 10.3389/fphys.2018.00878 (PMC6083813; doi:10.3389/fphys.2018.00878)
Supplement: Supplementary file 6 [file Data_Sheet_2.DOCX]

**Supplementary References: Publications used to construct T cell differentiation model**

Acosta-Rodriguez EV, Rivino L, Geginat J, Jarrossay D, Gattorno M, Lanzavecchia A, Sallusto F, and Napolitani G.Surface phenotype and antigenic specificity of human interleukin 17-producing T helper memory cells.Nat Immunol 2007 Jun8; (6) 639-46.pmid:17486092

Afkarian M, Sedy JR, Yang J, Jacobson NG, Cereb N, Yang SY, Murphy TL, and Murphy KM.T-bet is a STAT1-induced regulator of IL-12R expression in naïve CD4+ T cells.Nat Immunol 2002 Jun; 3(6) 549-57. pmid:12006974.

Akira S.The role of IL-18 in innate immunity.Curr Opin Immunol 2000 Feb12; (1) 59-63.pmid:10679398

Bettelli E, Dastrange M, and Oukka M.Foxp3 interacts with nuclear factor of activated T cells and NF-kappa B to repress cytokine gene expression and effector functions of T helper cells.Proc Natl Acad Sci U S A 2005 Apr 5; 102(14) 5138-43. pmid:15790681.

Bettelli E, Carrier Y, Gao W, Korn T, Strom TB, Oukka M, Weiner HL, and Kuchroo VK.Reciprocal developmental pathways for the generation of pathogenic effector TH17 and regulatory T cells.Nature 2006 May 11441; (7090) 235-8.pmid:16648838

Burchill MA, Yang J, Vogtenhuber C, Blazar BR, and Farrar MA.IL-2 receptor beta-dependent STAT5 activation is required for the development of Foxp3+ regulatory T cells.J Immunol 2007 Jan 1178; (1) 280-90.pmid:17182565

Carbo A, Hontecillas R, Kronsteiner B, Viladomiu M, Pedragosa M, Lu P, Philipson CW, Hoops S, Marathe M, Eubank S, Bisset K, Wendelsdorf K, Jarrah A, Mei Y, and Bassaganya-Riera J.Systems modeling of molecular mechanisms controlling cytokine-driven CD4+ T cell differentiation and phenotype plasticity.PLoS Comput Biol 2013 Apr9; (4) e1003027.pmid:23592971

Chang JT, Segal BM, Nakanishi K, Okamura H, and Shevach EM.The costimulatory effect of IL-18 on the induction of antigen-specific IFN-gamma production by resting T cells is IL-12 dependent and is mediated by up-regulation of the IL-12 receptor beta2 subunit.Eur J Immunol 2000 Apr30; (4) 1113-9.pmid:10760800

Chen W, Jin W, Hardegen N, Lei KJ, Li L, Marinos N, McGrady G, and Wahl SM.Conversion of peripheral CD4+CD25- naive T cells to CD4+CD25+ regulatory T cells by TGF-beta induction of transcription factor Foxp3.J Exp Med 2003 Dec 15198; (12) 1875-86.pmid:14676299

Chen Z, Laurence A, Kanno Y, Pacher-Zavisin M, Zhu BM, Tato C, Yoshimura A, Hennighausen L, and O'Shea JJ.Selective regulatory function of Socs3 in the formation of IL-17-secreting T cells.Proc Natl Acad Sci U S A 2006 May 23103; (21) 8137-42.pmid:16698929

Chen Z, Lund R, Aittokallio T, Kosonen M, Nevalainen O, and Lahesmaa R.Identification of novel IL-4/Stat6-regulated genes in T lymphocytes.J Immunol 2003 Oct 1171; (7) 3627-35.pmid:14500660

Cooney RN.Suppressors of cytokine signaling (SOCS): inhibitors of the JAK/STAT pathway.Shock 2002 Feb17; (2) 83-90.pmid:11837794

Cote-Sierra J, Foucras G, Guo L, Chiodetti L, Young HA, Hu-Li J, Zhu J, and Paul WE.Interleukin 2 plays a central role in Th2 differentiation.Proc Natl Acad Sci U S A 2004 Mar 16101; (11) 3880-5.pmid:15004274

Dardalhon V, Awasthi A, Kwon H, Galileos G, Gao W, Sobel RA, Mitsdoerffer M, Strom TB, Elyaman W, Ho IC, Khoury S, Oukka M, and Kuchroo VK.IL-4 inhibits TGF-beta-induced Foxp3+ T cells and, together with TGF-beta, generates IL-9+ IL-10+ Foxp3(-) effector T cells.Nat Immunol 2008 Dec9; (12) 1347-55.pmid:18997793

Diehl S, Anguita J, Hoffmeyer A, Zapton T, Ihle JN, Fikrig E, and Rincón M.Inhibition of Th1 differentiation by IL-6 is mediated by SOCS1.Immunity 2000 Dec13; (6) 805-15.pmid:11163196

Elser B, Lohoff M, Kock S, Giaisi M, Kirchhoff S, Krammer PH, and Li-Weber M.IFN-gamma represses IL-4 expression via IRF-1 and IRF-2.Immunity 2002 Dec17; (6) 703-12.pmid:12479817

Evans CM and Jenner RG.Transcription factor interplay in T helper cell differentiation.Brief Funct Genomics 2013 Nov12; (6) 499-511.pmid:23878131

Fantini MC, Becker C, Monteleone G, Pallone F, Galle PR, and Neurath MF.Cutting edge: TGF-beta induces a regulatory phenotype in CD4+CD25- T cells through Foxp3 induction and down-regulation of Smad7.J Immunol 2004 May 1172; (9) 5149-53.pmid:15100250

Fu S, Zhang N, Yopp AC, Chen D, Mao M, Chen D, Zhang H, Ding Y, and Bromberg JS.TGF-beta induces Foxp3 + T-regulatory cells from CD4 + CD25 - precursors.Am J Transplant 2004 Oct4; (10) 1614-27.pmid:15367216

Furuta S, Kagami S, Tamachi T, Ikeda K, Fujiwara M, Suto A, Hirose K, Watanabe N, Saito Y, Iwamoto I, and Nakajima H.Overlapping and distinct roles of STAT4 and T-bet in the regulation of T cell differentiation and allergic airway inflammation.J Immunol 2008 May 15180; (10) 6656-62.pmid:18453585

Gavin MA, Rasmussen JP, Fontenot JD, Vasta V, Manganiello VC, Beavo JA, and Rudensky AY.Foxp3-dependent programme of regulatory T-cell differentiation.Nature 2007 Feb 15445; (7129) 771-5.pmid:17220874

Gocke AR, Cravens PD, Ben LH, Hussain RZ, Northrop SC, Racke MK, and Lovett-Racke AE.T-bet regulates the fate of Th1 and Th17 lymphocytes in autoimmunity.J Immunol 2007 Feb 1178; (3) 1341-8.pmid:17237380

Goodbourn S, Didcock L, and Randall RE.Interferons: cell signalling, immune modulation, antiviral response and virus countermeasures.J Gen Virol 2000 Oct81; (Pt 10) 2341-64.pmid:10993923

Gorelik L, Fields PE, and Flavell RA.Cutting edge: TGF-beta inhibits Th type 2 development through inhibition of GATA-3 expression.J Immunol 2000 Nov 1165; (9) 4773-7.pmid:11045997

Greenlund AC, Farrar MA, Viviano BL, and Schreiber RD.Ligand-induced IFN gamma receptor tyrosine phosphorylation couples the receptor to its signal transduction system (p91).EMBO J 1994 Apr 113; (7) 1591-600.pmid:8156998

Hebenstreit D, Luft P, Schmiedlechner A, Duschl A, and Horejs-Hoeck J.SOCS-1 and SOCS-3 inhibit IL-4 and IL-13 induced activation of Eotaxin-3/CCL26 gene expression in HEK293 cells.Mol Immunol 2005 Feb42; (3) 295-303.pmid:15589317

Heinrich PC, Behrmann I, Müller-Newen G, Schaper F, and Graeve L.Interleukin-6-type cytokine signalling through the gp130/Jak/STAT pathway.Biochem J 1998 Sep 1334 ( Pt 2); 297-314.pmid:9716487

Huang Z, Xin J, Coleman J, and Huang H.IFN-gamma suppresses STAT6 phosphorylation by inhibiting its recruitment to the IL-4 receptor.J Immunol 2005 Feb 1174; (3) 1332-7.pmid:15661890

Hwang ES, Hong JH, and Glimcher LH.IL-2 production in developing Th1 cells is regulated by heterodimerization of RelA and T-bet and requires T-bet serine residue 508.J Exp Med 2005 Nov 7202; (9) 1289-300.pmid:16275766

Hwang ES, Szabo SJ, Schwartzberg PL, and Glimcher LH.T helper cell fate specified by kinase-mediated interaction of T-bet with GATA-3.Science 2005 Jan 21307; (5708) 430-3.pmid:15662016

Ichiyama K, Yoshida H, Wakabayashi Y, Chinen T, Saeki K, Nakaya M, Takaesu G, Hori S, Yoshimura A, and Kobayashi T.Foxp3 inhibits RORgammat-mediated IL-17A mRNA transcription through direct interaction with RORgammat.J Biol Chem 2008 Jun 20283; (25) 17003-8.pmid:18434325

Igarashi K, Garotta G, Ozmen L, Ziemiecki A, Wilks AF, Harpur AG, Larner AC, and Finbloom DS.Interferon-gamma induces tyrosine phosphorylation of interferon-gamma receptor and regulated association of protein tyrosine kinases, Jak1 and Jak2, with its receptor.J Biol Chem 1994 May 20; 269(20) 14333-6. pmid:7514165.

Ivanov II, McKenzie BS, Zhou L, Tadokoro CE, Lepelley A, Lafaille JJ, Cua DJ, and Littman DR.The orphan nuclear receptor RORgammat directs the differentiation program of proinflammatory IL-17+ T helper cells.Cell 2006 Sep 22126; (6) 1121-33.pmid:16990136

Iwakura Y and Ishigame H.The IL-23/IL-17 axis in inflammation.J Clin Invest 2006 May116; (5) 1218-22.pmid:16670765

Kamiya S, Owaki T, Morishima N, Fukai F, Mizuguchi J, and Yoshimoto T.An indispensable role for STAT1 in IL-27-induced T-bet expression but not proliferation of naive CD4+ T cells.J Immunol 2004 Sep 15173; (6) 3871-7.pmid:15356135

Kaplan MH, Schindler U, Smiley ST, and Grusby MJ.Stat6 is required for mediating responses to IL-4 and for development of Th2 cells.Immunity 1996 Mar4; (3) 313-9.pmid:8624821

Kimura A, Naka T, Nagata S, Kawase I, and Kishimoto T.SOCS-1 suppresses TNF-alpha-induced apoptosis through the regulation of Jak activation.Int Immunol 2004 Jul16; (7) 991-9.pmid:15173123

Kishimoto T, Akira S, and Taga T.Interleukin-6 and its receptor: a paradigm for cytokines.Science 1992 Oct 23258; (5082) 593-7.pmid:1411569

Korn T, Bettelli E, Gao W, Awasthi A, Jäger A, Strom TB, Oukka M, and Kuchroo VK.IL-21 initiates an alternative pathway to induce proinflammatory T(H)17 cells.Nature 2007 Jul 26; 448(7152) 484-7. pmid:17581588.

Kotenko SV and Pestka S.Jak-Stat signal transduction pathway through the eyes of cytokine class II receptor complexes.Oncogene 2000 May 1519; (21) 2557-65.pmid:10851054

Krause CD, He W, Kotenko S, and Pestka S.Modulation of the activation of Stat1 by the interferon-gamma receptor complex.Cell Res 2006 Jan16; (1) 113-23.pmid:16467883

Kurebayashi Y, Nagai S, Ikejiri A, and Koyasu S.Recent advances in understanding the molecular mechanisms of the development and function of Th17 cells.Genes Cells 2013 Apr18; (4) 247-65.pmid:23383714

Langrish CL, Chen Y, Blumenschein WM, Mattson J, Basham B, Sedgwick JD, McClanahan T, Kastelein RA, and Cua DJ.IL-23 drives a pathogenic T cell population that induces autoimmune inflammation.J Exp Med 2005 Jan 17201; (2) 233-40.pmid:15657292

Laurence A, Tato CM, Davidson TS, Kanno Y, Chen Z, Yao Z, Blank RB, Meylan F, Siegel R, Hennighausen L, Shevach EM, and O'shea JJ.Interleukin-2 signaling via STAT5 constrains T helper 17 cell generation.Immunity 2007 Mar26; (3) 371-81.pmid:17363300

Lazarevic V, Glimcher LH, and Lord GM.T-bet: a bridge between innate and adaptive immunity.Nat Rev Immunol 2013 Nov13; (11) 777-89.pmid:24113868

Lighvani AA, Frucht DM, Jankovic D, Yamane H, Aliberti J, Hissong BD, Nguyen BV, Gadina M, Sher A, Paul WE, and O'Shea JJ.T-bet is rapidly induced by interferon-gamma in lymphoid and myeloid cells.Proc Natl Acad Sci U S A 2001 Dec 1898; (26) 15137-42.pmid:11752460

Losman JA, Chen XP, Hilton D, and Rothman P.Cutting edge: SOCS-1 is a potent inhibitor of IL-4 signal transduction.J Immunol 1999 Apr 1; 162(7) 3770-4. pmid:10201892.

Losman JA, Chen XP, Hilton D, and Rothman P.Cutting edge: SOCS-1 is a potent inhibitor of IL-4 signal transduction.J Immunol 1999 Apr 1162; (7) 3770-4.pmid:10201892

Macian F.NFAT proteins: key regulators of T-cell development and function.Nat Rev Immunol 2005 Jun; 5(6) 472-84. pmid:15928679.

Malek TR.The biology of interleukin-2.Annu Rev Immunol 200826; 453-79.pmid:18062768

Mantel PY, Kuipers H, Boyman O, Rhyner C, Ouaked N, Rückert B, Karagiannidis C, Lambrecht BN, Hendriks RW, Crameri R, Akdis CA, Blaser K, and Schmidt-Weber CB.GATA3-driven Th2 responses inhibit TGF-beta1-induced FOXP3 expression and the formation of regulatory T cells.PLoS Biol 2007 Dec5; (12) e329.pmid:18162042

Mathur AN, Chang HC, Zisoulis DG, Stritesky GL, Yu Q, O'Malley JT, Kapur R, Levy DE, Kansas GS, and Kaplan MH.Stat3 and Stat4 direct development of IL-17-secreting Th cells.J Immunol 2007 Apr 15178; (8) 4901-7.pmid:17404271

McGeachy MJ, Chen Y, Tato CM, Laurence A, Joyce-Shaikh B, Blumenschein WM, McClanahan TK, O'Shea JJ, and Cua DJ.The interleukin 23 receptor is essential for the terminal differentiation of interleukin 17-producing effector T helper cells in vivo.Nat Immunol 2009 Mar10; (3) 314-24.pmid:19182808

Mendoza L and Pardo F.A robust model to describe the differentiation of T-helper cells.Theory Biosci 2010 Dec129; (4) 283-93.pmid:20922578

Mendoza L.A network model for the control of the differentiation process in Th cells.Biosystems 2006 May84; (2) 101-14.pmid:16386358

Mullen AC, High FA, Hutchins AS, Lee HW, Villarino AV, Livingston DM, Kung AL, Cereb N, Yao TP, Yang SY, and Reiner SL.Role of T-bet in commitment of TH1 cells before IL-12-dependent selection.Science 2001 Jun 8; 292(5523) 1907-10. pmid:11397944.

Murawski MR, Litherland SA, Clare-Salzler MJ, and Davoodi-Semiromi A.Upregulation of Foxp3 expression in mouse and human Treg is IL-2/STAT5 dependent: implications for the NOD STAT5B mutation in diabetes pathogenesis.Ann N Y Acad Sci 2006 Oct1079; 198-204.pmid:17130555

Murphy KM and Reiner SL.The lineage decisions of helper T cells.Nat Rev Immunol 2002 Dec2; (12) 933-44.pmid:12461566

Nakanishi K, Yoshimoto T, Tsutsui H, and Okamura H.Interleukin-18 is a unique cytokine that stimulates both Th1 and Th2 responses depending on its cytokine milieu.Cytokine Growth Factor Rev 2001 Mar12; (1) 53-72.pmid:11312119

Novick D, Kim S, Kaplanski G, and Dinarello CA.Interleukin-18, more than a Th1 cytokine.Semin Immunol 2013 Dec 1525; (6) 439-48.pmid:24275602

Nurieva R, Yang XO, Martinez G, Zhang Y, Panopoulos AD, Ma L, Schluns K, Tian Q, Watowich SS, Jetten AM, and Dong C.Essential autocrine regulation by IL-21 in the generation of inflammatory T cells.Nature 2007 Jul 26448; (7152) 480-3.pmid:17581589

O'Shea JJ, Steward-Tharp SM, Laurence A, Watford WT, Wei L, Adamson AS, and Fan S.Signal transduction and Th17 cell differentiation.Microbes Infect 2009 Apr11; (5) 599-611.pmid:19379825

Ouyang W, Löhning M, Gao Z, Assenmacher M, Ranganath S, Radbruch A, and Murphy KM.Stat6-independent GATA-3 autoactivation directs IL-4-independent Th2 development and commitment.Immunity 2000 Jan12; (1) 27-37.pmid:10661403

Owaki T, Asakawa M, Morishima N, Hata K, Fukai F, Matsui M, Mizuguchi J, and Yoshimoto T.A role for IL-27 in early regulation of Th1 differentiation.J Immunol 2005 Aug 15175; (4) 2191-200.pmid:16081786

Parham C, Chirica M, Timans J, Vaisberg E, Travis M, Cheung J, Pflanz S, Zhang R, Singh KP, Vega F, To W, Wagner J, O'Farrell AM, McClanahan T, Zurawski S, Hannum C, Gorman D, Rennick DM, Kastelein RA, de Waal Malefyt R, and Moore KW.A receptor for the heterodimeric cytokine IL-23 is composed of IL-12Rbeta1 and a novel cytokine receptor subunit, IL-23R.J Immunol 2002 Jun 1168; (11) 5699-708.pmid:12023369

Park H, Li Z, Yang XO, Chang SH, Nurieva R, Wang YH, Wang Y, Hood L, Zhu Z, Tian Q, and Dong C.A distinct lineage of CD4 T cells regulates tissue inflammation by producing interleukin 17.Nat Immunol 2005 Nov6; (11) 1133-41.pmid:16200068

Park WR, Nakahira M, Sugimoto N, Bian Y, Yashiro-Ohtani Y, Zhou XY, Yang YF, Hamaoka T, and Fujiwara H.A mechanism underlying STAT4-mediated up-regulation of IFN-gamma induction inTCR-triggered T cells.Int Immunol 2004 Feb; 16(2) 295-302. pmid:14734615.

Passerini L, Allan SE, Battaglia M, Di Nunzio S, Alstad AN, Levings MK, Roncarolo MG, and Bacchetta R.STAT5-signaling cytokines regulate the expression of FOXP3 in CD4+CD25+ regulatory T cells and CD4+CD25- effector T cells.Int Immunol 2008 Mar20; (3) 421-31.pmid:18270368

Ranganath S and Murphy KM.Structure and specificity of GATA proteins in Th2 development.Mol Cell Biol 2001 Apr21; (8) 2716-25.pmid:11283251

Robinson D, Shibuya K, Mui A, Zonin F, Murphy E, Sana T, Hartley SB, Menon S, Kastelein R, Bazan F, and O'Garra A.IGIF does not drive Th1 development but synergizes with IL-12 for interferon-gamma production and activates IRAK and NFkappaB.Immunity 1997 Oct7; (4) 571-81.pmid:9354477

Rudensky AY, Gavin M, and Zheng Y.FOXP3 and NFAT: partners in tolerance.Cell 2006 Jul 28126; (2) 253-6.pmid:16873058

Saito H, Morita Y, Fujimoto M, Narazaki M, Naka T, and Kishimoto T.IFN regulatory factor-1-mediated transcriptional activation of mouse STAT-induced STAT inhibitor-1 gene promoter by IFN-gamma.J Immunol 2000 Jun 1164; (11) 5833-43.pmid:10820262

Sato T, Saito R, Jinushi T, Tsuji T, Matsuzaki J, Koda T, Nishimura Si, Takeshima H, and Nishimura T.IFN-gamma-induced SOCS-1 regulates STAT6-dependent eotaxin production triggered by IL-4 and TNF-alpha.Biochem Biophys Res Commun 2004 Feb 6314; (2) 468-75.pmid:14733929

Scheinman EJ and Avni O.Transcriptional regulation of GATA3 in T helper cells by the integrated activities of transcription factors downstream of the interleukin-4 receptor and T cell receptor.J Biol Chem 2009 Jan 30284; (5) 3037-48.pmid:19056736

Shevach EM, DiPaolo RA, Andersson J, Zhao DM, Stephens GL, and Thornton AM.The lifestyle of naturally occurring CD4+ CD25+ Foxp3+ regulatory T cells.Immunol Rev 2006 Aug212; 60-73.pmid:16903906

Smeltz RB, Chen J, Hu-Li J, and Shevach EM.Regulation of interleukin (IL)-18 receptor alpha chain expression on CD4(+) T cells during T helper (Th)1/Th2 differentiation. Critical downregulatory role of IL-4.J Exp Med 2001 Jul 16194; (2) 143-53.pmid:11457889

Smits HH, van Rietschoten JG, Hilkens CM, Sayilir R, Stiekema F, Kapsenberg ML, and Wierenga EA.IL-12-induced reversal of human Th2 cells is accompanied by full restoration of IL-12 responsiveness and loss of GATA-3 expression.Eur J Immunol 2001 Apr31; (4) 1055-65.pmid:11298330

Song MM and Shuai K.The suppressor of cytokine signaling (SOCS) 1 and SOCS3 but not SOCS2 proteins inhibit interferon-mediated antiviral and antiproliferative activities.J Biol Chem 1998 Dec 25273; (52) 35056-62.pmid:9857039

Spolski R and Leonard WJ.Interleukin-21: a double-edged sword with therapeutic potential.Nat Rev Drug Discov 2014 May13; (5) 379-95.pmid:24751819

Spolski R and Leonard WJ.Interleukin-21: basic biology and implications for cancer and autoimmunity.Annu Rev Immunol 200826; 57-79.pmid:17953510

Stumhofer JS, Silver J, and Hunter CA.Negative regulation of Th17 responses.Semin Immunol 2007 Dec19; (6) 394-9.pmid:18221887

Swain SL.Interleukin 18: tipping the balance towards a T helper cell 1 response.J Exp Med 2001 Aug 6194; (3) F11-4.pmid:11489958

Szabo SJ, Kim ST, Costa GL, Zhang X, Fathman CG, and Glimcher LH.A novel transcription factor, T-bet, directs Th1 lineage commitment.Cell 2000 Mar 17; 100(6) 655-69. pmid:10761931.

Szabo SJ, Kim ST, Costa GL, Zhang X, Fathman CG, and Glimcher LH.A novel transcription factor, T-bet, directs Th1 lineage commitment.Cell 2000 Mar 17100; (6) 655-69.pmid:10761931

Takeda K, Kaisho T, Yoshida N, Takeda J, Kishimoto T, and Akira S.Stat3 activation is responsible for IL-6-dependent T cell proliferation through preventing apoptosis: generation and characterization of T cell-specific Stat3-deficient mice.J Immunol 1998 Nov 1161; (9) 4652-60.pmid:9794394

Tamachi T, Takatori H, Fujiwara M, Hirose K, Maezawa Y, Kagami S, Suto A, Watanabe N, Iwamoto I, and Nakajima H.STAT6 inhibits T-bet-independent Th1 cell differentiation.Biochem Biophys Res Commun 2009 May 15382; (4) 751-5.pmid:19324016

Thierfelder WE, van Deursen JM, Yamamoto K, Tripp RA, Sarawar SR, Carson RT, Sangster MY, Vignali DA, Doherty PC, Grosveld GC, and Ihle JN.Requirement for Stat4 in interleukin-12-mediated responses of natural killer and T cells.Nature 1996 Jul 11382; (6587) 171-4.pmid:8700208

Tian Y and Zajac AJ.IL-21 and T Cell Differentiation: Consider the Context.Trends Immunol 2016 Aug37; (8) 557-68.pmid:27389961

Tominaga K, Yoshimoto T, Torigoe K, Kurimoto M, Matsui K, Hada T, Okamura H, and Nakanishi K.IL-12 synergizes with IL-18 or IL-1beta for IFN-gamma production from human T cells.Int Immunol 2000 Feb12; (2) 151-60.pmid:10653850

Torgerson TR, Genin A, Chen C, Zhang M, Zhou B, Añover-Sombke S, Frank MB, Dozmorov I, Ocheltree E, Kulmala P, Centola M, Ochs HD, Wells AD, and Cron RQ.FOXP3 inhibits activation-induced NFAT2 expression in T cells thereby limiting effector cytokine expression.J Immunol 2009 Jul 15183; (2) 907-15.pmid:19564342

Trinchieri G.Interleukin-12 and the regulation of innate resistance and adaptive immunity.Nat Rev Immunol 2003 Feb3; (2) 133-46.pmid:12563297

Usui T, Nishikomori R, Kitani A, and Strober W.GATA-3 suppresses Th1 development by downregulation of Stat4 and not through effects on IL-12Rbeta2 chain or T-bet.Immunity 2003 Mar18; (3) 415-28.pmid:12648458

Usui T, Preiss JC, Kanno Y, Yao ZJ, Bream JH, O'Shea JJ, and Strober W.T-bet regulates Th1 responses through essential effects on GATA-3 function rather than on IFNG gene acetylation and transcription.J Exp Med 2006 Mar 20203; (3) 755-66.pmid:16520391

Veldhoen M, Hocking RJ, Atkins CJ, Locksley RM, and Stockinger B.TGFbeta in the context of an inflammatory cytokine milieu supports de novo differentiation of IL-17-producing T cells.Immunity 2006 Feb24; (2) 179-89.pmid:16473830

Venkataraman C, Leung S, Salvekar A, Mano H, and Schindler U.Repression of IL-4-induced gene expression by IFN-gamma requires Stat1 activation.J Immunol 1999 Apr 1162; (7) 4053-61.pmid:10201928

Wan YY and Flavell RA.Regulatory T-cell functions are subverted and converted owing to attenuated Foxp3 expression.Nature 2007 Feb 15445; (7129) 766-70.pmid:17220876

Weaver CT and Hatton RD.Interplay between the TH17 and TReg cell lineages: a (co-)evolutionary perspective.Nat Rev Immunol 2009 Dec9; (12) 883-9.pmid:19935807

Wei L, Laurence A, Elias KM, and O'Shea JJ.IL-21 is produced by Th17 cells and drives IL-17 production in a STAT3-dependent manner.J Biol Chem 2007 Nov 30282; (48) 34605-10.pmid:17884812

Williams LM and Rudensky AY.Maintenance of the Foxp3-dependent developmental program in mature regulatory T cells requires continued expression of Foxp3.Nat Immunol 2007 Mar8; (3) 277-84.pmid:17220892

Wurster AL, Withers DJ, Uchida T, White MF, and Grusby MJ.Stat6 and IRS-2 cooperate in interleukin 4 (IL-4)-induced proliferation and differentiation but are dispensable for IL-4-dependent rescue from apoptosis.Mol Cell Biol 2002 Jan22; (1) 117-26.pmid:11739727

Yang XP, Ghoreschi K, Steward-Tharp SM, Rodriguez-Canales J, Zhu J, Grainger JR, Hirahara K, Sun HW, Wei L, Vahedi G, Kanno Y, O'Shea JJ, and Laurence A.Opposing regulation of the locus encoding IL-17 through direct, reciprocal actions of STAT3 and STAT5.Nat Immunol 2011 Mar12; (3) 247-54.pmid:21278738

Yao Z, Kanno Y, Kerenyi M, Stephens G, Durant L, Watford WT, Laurence A, Robinson GW, Shevach EM, Moriggl R, Hennighausen L, Wu C, and O'Shea JJ.Nonredundant roles for Stat5a/b in directly regulating Foxp3.Blood 2007 May 15; 109(10) 4368-75. pmid:17227828.

Yoshimoto T, Takeda K, Tanaka T, Ohkusu K, Kashiwamura S, Okamura H, Akira S, and Nakanishi K.IL-12 up-regulates IL-18 receptor expression on T cells, Th1 cells, and B cells: synergism with IL-18 for IFN-gamma production.J Immunol 1998 Oct 1161; (7) 3400-7.pmid:9759857

Yu CR, Mahdi RM, Ebong S, Vistica BP, Chen J, Guo Y, Gery I, and Egwuagu CE.Cell proliferation and STAT6 pathways are negatively regulated in T cells by STAT1 and suppressors of cytokine signaling.J Immunol 2004 Jul 15; 173(2) 737-46. pmid:15240659.

Zhou L, Chong MM, and Littman DR.Plasticity of CD4+ T cell lineage differentiation.Immunity 2009 May30; (5) 646-55.pmid:19464987

Zhou L, Lopes JE, Chong MM, Ivanov II, Min R, Victora GD, Shen Y, Du J, Rubtsov YP, Rudensky AY, Ziegler SF, and Littman DR.TGF-beta-induced Foxp3 inhibits T(H)17 cell differentiation by antagonizing RORgammat function.Nature 2008 May 8453; (7192) 236-40.pmid:18368049

Zhou M and Ouyang W.The function role of GATA-3 in Th1 and Th2 differentiation.Immunol Res 200328; (1) 25-37.pmid:12947222

Zhou M, Ouyang W, Gong Q, Katz SG, White JM, Orkin SH, and Murphy KM.Friend of GATA-1 represses GATA-3-dependent activity in CD4+ T cells.J Exp Med 2001 Nov 19194; (10) 1461-71.pmid:11714753

Zhu J, Cote-Sierra J, Guo L, and Paul WE.Stat5 activation plays a critical role in Th2 differentiation.Immunity 2003 Nov19; (5) 739-48.pmid:14614860

Zhu J, Yamane H, and Paul WE.Differentiation of effector CD4 T cell populations (*).Annu Rev Immunol 201028; 445-89.pmid:20192806

Zhu J, Yamane H, Cote-Sierra J, Guo L, and Paul WE.GATA-3 promotes Th2 responses through three different mechanisms: induction of Th2 cytokine production, selective growth of Th2 cells and inhibition of Th1 cell-specific factors.Cell Res 2006 Jan; 16(1) 3-10. pmid:16467870.
