# Supplementary material for: A Mechanistic Computational Model Reveals That Plasticity of CD4+ T Cell Differentiation Is a Function of Cytokine Composition and Dosage
Source: Front Physiol. 2018 Aug 2;9:878. doi: 10.3389/fphys.2018.00878 (PMC6083813; doi:10.3389/fphys.2018.00878)
Supplement: Supplementary file 7 [file Data_Sheet_3.DOCX]

Supplementary Material

Plasticity of CD4^+^ T cell differentiation is a function of cytokine composition and dosage

Bhanwar Lal Puniya, Robert G Todd*, Akram Mohammed, Deborah M. Brown, Matteo Barberis*, Tomáš Helikar^*^

*** Correspondence:** Corresponding Authors: [thelikar2@unl.edu](mailto:thelikar2@unl.edu); [rtodd@mtmercy.edu](mailto:rtodd@mtmercy.edu); [M.Barberis@uva.nl](mailto:M.Barberis@uva.nl)

Supplementary Figures and Tables

## Supplementary Figures


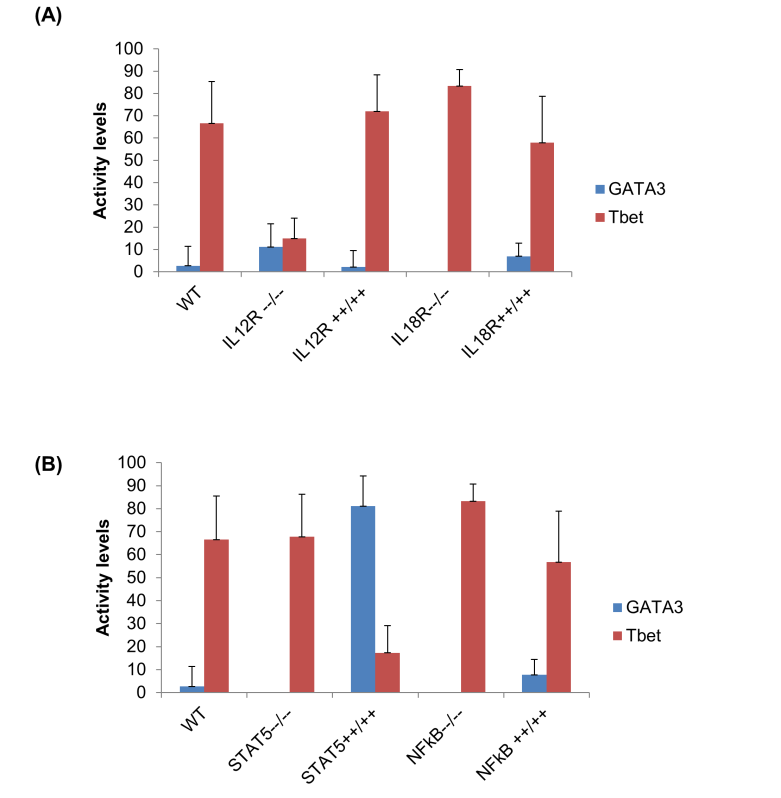


**Supplementary Figure 1.** **Combination of IL-12 and IL-18 in the presence of IL-6 favors Th1-Th2 phenotype in NF-κB and STAT5 dependent manner.**

(A) The model was simulated using input composition (TCR ligand + IL-12 + IL-18 + IL-6) under five conditions, including wild type, and knock-out and overexpression of both IL-12R and IL-18R. Over-expression of IL-12R favors Th1 phenotype, whereas IL-12R knock-out significantly increased the activity level of GATA3. IL-18R knock-out completely inactivated both GATA3 and Foxp3 (B) Knock-out of STAT5 and NF-κB resulted in complete inactivation of GATA3, whereas overexpression of STAT5 induced strong GATA3 response. Overexpression of NF-κB increased activity of GATA3.

## Supplementary Tables (Captions)

**Supplementary Table 1: Classical T-cell differentiation behaviors reproduced by model**

**Supplementary Table 2: Input compositions of all the T cell phenotypes**

**Supplementary Table 3: Minimal and maximal input composition for all T cell phenotypes**

**Supplementary Table 4: Sensitivity analysis results under minimal and maximal input compositions for all the phenotypes**
